# Multihost *Bartonella* parasites display covert host specificity even when transmitted by generalist vectors

**DOI:** 10.1111/1365-2656.12568

**Published:** 2016-08-16

**Authors:** Susan M. Withenshaw, Godefroy Devevey, Amy B. Pedersen, Andy Fenton

**Affiliations:** ^1^ Institute of Integrative BiologyUniversity of LiverpoolCrown StreetLiverpoolMerseysideL69 7ZBUK; ^2^ NERC Centre for Ecology and HydrologyMaclean BuildingBenson LaneCrowmarsh GiffordOxfordshireOX10 8BBUK; ^3^ School of Biology & Centre for ImmunityInfection and EvolutionUniversity of EdinburghAshworth LaboratoriesCharlotte Auerbach RoadEdinburghEH9 3FLUK

**Keywords:** *Apodemus sylvaticus*, *Bartonella*, fleas, host‐generalist, host‐specialist, *Myodes glareolus*, pathogen genotypes, rodents, sequencing, vector‐borne diseases

## Abstract

1. Many parasites infect multiple sympatric host species, and there is a general assumption that parasite transmission between co‐occurring host species is commonplace. Such between‐species transmission could be key to parasite persistence within a disease reservoir and is consequently an emerging focus for disease control.
2. However, while a growing body of theory indicates the potential importance of between‐species transmission for parasite persistence, conclusive empirical evidence from natural communities is lacking, and the assumption that between‐species transmission is inevitable may therefore be wrong.
3. We investigated the occurrence of between‐species transmission in a well‐studied multihost parasite system. We identified the flea‐borne *Bartonella* parasites infecting sympatric populations of *Apodemus sylvaticus* (wood mice) and *Myodes glareolus* (bank voles) in the UK and confirmed that several *Bartonella* species infect both rodent species. However, counter to previous knowledge, genetic characterization of these parasites revealed covert host specificity, where each host species is associated with a distinct assemblage of genetic variants, indicating that between‐species transmission is rare.
4. Limited between‐species transmission could result from rare encounters between one host species and the parasites infecting another and/or host–parasite incompatibility. We investigated the occurrence of such encounter and compatibility barriers by identifying the flea species associated with each rodent host, and the *Bartonella* variants carried by individual fleas. We found that the majority of fleas were host‐generalists but the assemblage of *Bartonella* variants in fleas tended to reflect the assemblage of *Bartonella* variants in the host species they were collected from, thus providing evidence of encounter barriers mediated by limited between‐species flea transfer. However, we also found several fleas that were carrying variants never found in the host species from which they were collected, indicating some degree of host–pathogen incompatibility when barriers to encounter are overcome.
5. Overall, these findings challenge our default perceptions of multihost parasite persistence, as they show that despite considerable overlaps in host species ecology, separate populations of the same parasite species may circulate and persist independently in different sympatric host species. This questions our fundamental understanding of endemic transmission dynamics and the control of infection within natural reservoir communities.

## Introduction

Most parasites are able to infect multiple host species (Cleaveland, Laurenson & Taylor 2001; Woolhouse, Taylor & Haydon 2001), a realization that has fundamentally changed how we approach issues of disease control. This is because the endemic persistence of such ‘multihost’ parasites in wild host populations may rely on transmission between individuals of different host species (between‐species transmission) as well as, or even instead of, transmission between conspecifics (within‐species transmission) (Haydon *et al*. 2002; Holt *et al*. 2003; Dobson 2004; Fenton & Pedersen 2005; Streicker, Fenton & Pedersen 2013; Fenton *et al*. 2015). Consequently, successful control of infection in one host species may require interventions (e.g. vaccination or cullings) that target other species that dominate transmission in the host community (Laurenson *et al*. 2003; Donnelly *et al*. 2006; Serrano *et al*. 2011).

However, while a growing body of theory indicates the potential importance of between‐species transmission for endemic multihost parasite persistence (Holt & Pickering 1985; Bowers & Begon 1991; Begon *et al*. 1992; Bowers & Turner 1997; Greenman & Hudson 1999, 2000; Haydon *et al*. 2002; Holt *et al*. 2003; Dobson 2004; Fenton & Pedersen 2005), conclusive empirical evidence from natural communities is often lacking. The occurrence of between‐species transmission is often just assumed given that a parasite infects multiple sympatric host species (Dobson & Meagher 1996), or is concluded on the basis of indirect evidence such as correlations between parasite prevalence in one host species and population densities of another (Telfer *et al*. 2007a). However, such correlations may arise as a result of other processes not related to between‐species transmission, and therefore, the general importance of between‐species transmission in endemic parasite persistence in nature remains largely unknown.

The study of parasite genetics in wild communities represents an important means to address this knowledge gap (Streicker *et al*. 2010; Forrester & Hall 2014). Fine‐scale genetic characterization of multihost parasites may uncover structure within a parasite population that can provide direct evidence of the occurrence of between‐species transmission. Intriguingly, of the relatively few studies that have employed such techniques, many have found that sympatric host species are infected with different genetic variants of the same parasite species (Sehgal *et al*. 2006; Whiteman *et al*. 2006; Martínez‐Aquino *et al*. 2009). Such ‘covert host specificity’ indicates that discrete subsets of the same parasite species can circulate independently and persist within populations of sympatric host species with little or no between‐species transmission. This fundamentally challenges our default perceptions of endemic multihost parasite persistence, and it is therefore crucial to determine whether covert host specificity is a widespread phenomenon.

A lack of transmission between co‐occurring host species may result from limited between‐species contact opportunities and/or physiological incompatibility between variants and host species (‘encounter’ and ‘compatibility’ barriers, respectively; Combes 2001). Encounter barriers may easily break down if contact rates increase but between‐species transmission will remain inhibited if host–parasite incompatibility persists. Identifying the primary drivers of current covert host specificity (i.e. whether it arises due to current limitations in contact or exposure, or due to current incompatibility between parasite and host) could therefore indicate how stable the host specificity is, and enable predictions of how rapidly transmission dynamics are likely to change given future alterations to interactions within the host community.

Wild rodent communities are commonly used as model systems in which to study parasite infection and transmission dynamics within natural settings (Begon *et al*. 1999; Telfer *et al*. 2007a,b; Knowles *et al*. 2013; Turner *et al*. 2014), and they have been the focus of much multihost parasite research (Begon *et al*. 1999; Carslake *et al*. 2006; Streicker, Fenton & Pedersen 2013; Fenton *et al*. 2015). In particular, several species of rodent *Bartonella* are considered model examples of endemic multihost parasites, as these bacterial flea‐borne haemoparasites are commonly found to infect several sympatric rodent species (Birtles *et al*. 2001; Telfer *et al*. 2007a; Paziewska *et al*. 2012). However, previous inferences of between‐species *Bartonella* transmission within rodent populations have relied on observed differences in prevalence across different host community compositions (Telfer *et al*. 2007a), and, importantly, the possibility of covert host specificity (discrete populations of host‐specific variants) has not been directly addressed. Where genetic variation in populations of rodent *Bartonella* has been described (e.g. Birtles *et al*. 2001; Inoue *et al*. 2008; Berglund *et al*. 2010; Paziewska *et al*. 2011; Kosoy, Hayman & Chan 2012), it has largely been compared across broad geographic regions, or interpreted in relation to within‐individual and within‐species infection dynamics. In contrast, such variation has been rarely discussed in the context of between‐species transmission and multihost parasite persistence (although see Paziewska *et al*. 2012).

The vector‐borne nature of rodent *Bartonella* transmission (Bown, Bennet & Begon 2004; Morick *et al*. 2010; Gutiérrez *et al*. 2015) allows an assessment of whether any covert host specificity arises through current encounter barriers to between‐species transmission (i.e. limited exposure of one rodent species to fleas from another species), or through host‐*Bartonella* incompatibility. Although some rodent fleas are known to display differential host preferences (Khokhlova *et al*. 2012), close overlap between the flea communities of sympatric rodent species has also been demonstrated (Harris *et al*. 2009), and many flea species are documented as being able to infest several host species (Marshall 1981). Even so, host‐generalist fleas may still present a barrier to between‐species *Bartonella* transmission, as the rate of movement between different host species is likely to depend on the frequency and nature of between‐host contacts (Krasnov & Khokhlova 2001) or rate of visitation to another host species’ burrow, given that flea dispersal rates are generally low (Marshall 1981; Krasnov 2008). As such, we do not currently know the extent to which flea biting behaviour acts as a barrier to between‐species parasite transmission.

Through the genetic characterization of *Bartonella* infections in wild sympatric populations of *Apodemus sylvaticus* (Linneaus, 1978) (wood mice) and *Myodes glareolus* (Schreber, 1780) (bank voles), we provide conclusive evidence of covert host specificity in this well‐studied parasite system and therefore highlight that between‐species transmission of multihost parasites is potentially more rare than previously expected. Additionally, through characterizing the communities of fleas associated with each host species, and identifying the genetic variants of *Bartonella* carried by individual fleas taken from the different host species, we show that while vectors of multihost parasites may be generalists, ecological opportunities for vector transfer between different host species may be rare and therefore still represent a major impediment to between‐species parasite transmission.

## Materials and methods

### Field Sampling

Wood mice and bank voles were trapped using Sherman live‐traps (Alana Ecology, UK; dimensions 8·9 cm × 7·6 cm × 22·9 cm) and monitored longitudinally during 2011 and 2012 at three woodland sites in north‐west England: Manor Wood (MW; N 53·3301°, E ‐3·0516°), Maresfield & Gordale woods (MFG; N 53·2729°, E ‐3·0615°) and Rode Hall (RH; N 53·1213°, E ‐2·2798°). When first captured, all rodents were given a subcutaneous electronic PIT tag (AVID MicroChips, Lewes, UK) enabling individual identification. A small blood sample (~25 μL) was taken from the tail tip of each individual at each monthly capture to assess *Bartonella* infection. Blood samples were centrifuged at 16 000 *g* for 10 min to separate blood pellets (containing cells) from sera. Pellets were then frozen at −20 °C until further processing. Further details of field methods are given in Appendix S1 (Supporting Information).

Fleas were collected from rodents at MFG and RH in 2012 and during further field sampling at these sites in 2013 and 2014. Fleas were also collected from rodents at a fourth nearby site, Haddon Wood (HW; N 53·2709°, E ‐3·0268°; ~1·6 km from MFG and ~52 km from RH) during 2012. Fleas were removed from individuals by brushing the fur over a water bath, then stored individually in 90% ethanol and identified to species using a morphological key (Whitaker 2007). Some rodents were exposed to insecticide treatment as part of a concurrent experiment, but excluding these animals did not qualitatively affect the results obtained (compare Tables S2 and S3) and so data from all animals are presented throughout the main text.

### Identification of *Bartonella* dna in rodents and Fleas

DNA was extracted from rodent blood pellets and individual fleas using standard protocols (Appendix S1). *Bartonella* DNA was detected by PCR targeting a partial region of the 16S–23S internal transcribed spacer (hereafter referred to as the pITS region) following standard methodology (Roux & Raoult 1995; Birtles *et al*. 2000; Houpikian & Raoult 2001; Telfer *et al*. 2005, 2007a,b). As a non‐coding region of DNA, the pITS region can withstand many point mutations and insertion/deletion events and varies in length between different species of *Bartonella* (Roux & Raoult 1995; Birtles *et al*. 2000; Houpikian & Raoult 2001). We therefore assigned a *Bartonella* species identity to positive samples by first determining the size of the pITS amplicon(s) present when run on an agarose gel. This initial step also allowed identification of *‘*coinfections’ (where multiple species of *Bartonella* were present in the same sample), which were visible as multiple bands of different size on the gel.

Further to this species‐level classification, we identified genetic variation within these *Bartonella* species groups by sequencing a random subset of pITS amplicons of each size from each host species and site (see Appendix S1 and Fig. S1 for methods and assessment of sampling bias). We also sequenced amplicons from all *Bartonella*‐positive, non‐coinfected fleas. Species classifications of variants were confirmed by identifying the validated *Bartonella* species in GenBank with which each shared highest percentage similarity. This process also allowed differentiation between pITS sequences that are similar in length but somewhat divergent, and therefore likely to represent different *Bartonella* species.

### Investigating Covert Host Specificity of *Bartonella* Infecting Wood Mice and Bank Voles

We investigated whether wood mice and bank voles were associated with significantly different assemblages of *Bartonella* parasites using linear discriminant analyses (LDA) in the ‘*MASS’* package of R (v2.14.2) (R Core Team 2015). This analysis tests whether individuals can be identified to host species based only on the identity of the *Bartonella* DNA they were carrying (Venables & Ripley 2002). First, a random 75% subset of the true host‐*Bartonella* associations were used to train a host assignment model, which was then used to predict the host identity of the remaining 25% of the data. This was repeated 1000 times, each with a randomly selected set of training data, and mean prediction success was calculated. We then determined the mean prediction success of 1000 models trained using data that simulated random distributions of parasites across host species. The prediction successes of these two sets of models were compared using a chi‐squared test to determine whether host–parasite associations varied significantly from random expectations.

This analysis was first conducted on assemblages of *Bartonella* DNA identified to species level according to length of the pITS region. It was then repeated using the subset of *Bartonella* DNA that was sequenced and identified to pITS variant level to see if this afforded greater power to discriminate between host species (thus indicating covert host specificity). The analyses used combined data from all woodland sites (results were consistent when data from each site were analysed separately; Table S5). *Bartonella* species or variants observed on <5 occasions were omitted, as inclusion of very rare species/variants introduced computational problems when performing model validation. Since host‐specific *Bartonella* species comprising a single pITS variant have no potential for covert specificity, but may influence the power of parasite assemblages to discriminate between host species, we checked whether LDA results were affected by the inclusion of these species by rerunning all species‐level and variant‐level LDAs using multihost *Bartonella* infections only (i.e. infections with *Bartonella grahamii*,* Bartonella taylorii* and *Bartonella birtlesii*). We also confirmed that none of the results were biased by any particular *Bartonella* species, or by repeat sampling of individual rodents (Table S6).

### Comparison of Flea Communities Associated with Wood Mice and Bank Voles

Opportunities for between‐species *Bartonella* transmission may be limited by strong host preferences of different flea species. We investigated this possibility by using an LDA, as described above, to assess the similarity of flea assemblages infecting wood mice and bank voles. Host assignment models were trained on the associations between host and flea species, and we verified that sampling of multiple fleas from individual rodents did not affect the results (Table S7).

### Investigating Potential Flea Transfer Between Wood Mice and Bank Voles

In the absence of strong host preferences, fleas may still limit opportunities for between‐species *Bartonella* transmission if individual fleas rarely disperse between different host species. We therefore sought evidence of structure within the flea community that could indicate a general lack of movement/transfer between host species. We used an LDA, as described above, to determine whether the species identity of the host from which a flea was taken could be predicted based only on the *Bartonella* variant carried by a flea (results were not biased by any particular flea species, or by sampling of multiple flea specimens from individual rodents; Table S10). We also sought specific cases where fleas carried *Bartonella* variants never detected in the host species from which they were collected. Such occurrences would be evidence of host exposure to *Bartonella* variants from another host species but lack of infection, so suggesting the presence of a host–parasite compatibility barrier rather than a lack of ecological opportunity for infection. Since the host specificity of *Bartonella* variants was determined from data collected in 2011 and 2012, whereas fleas were collected from hosts during 2012–2014 and at an additional site (HW), we checked for the consistency of these results using only data for which the characterization of *Bartonella* DNA in rodents and fleas at the same sites and in the same sampling year was available (i.e. MFG and RH in 2012).

## Results

### *Bartonella* in rodents: overall prevalence

Blood samples were taken from 743 wood mice (1376 samples) and 751 bank voles (1224 samples). *Bartonella* DNA was detected in 816 (59·3%) wood mouse and 599 (48·9%) bank vole samples. *Bartonella* coinfections were detected in 23·2% of positive samples from wood mice and 15·2% of positive samples from bank voles.

### *Bartonella* in rodents: species‐level data

**Figure 1:**
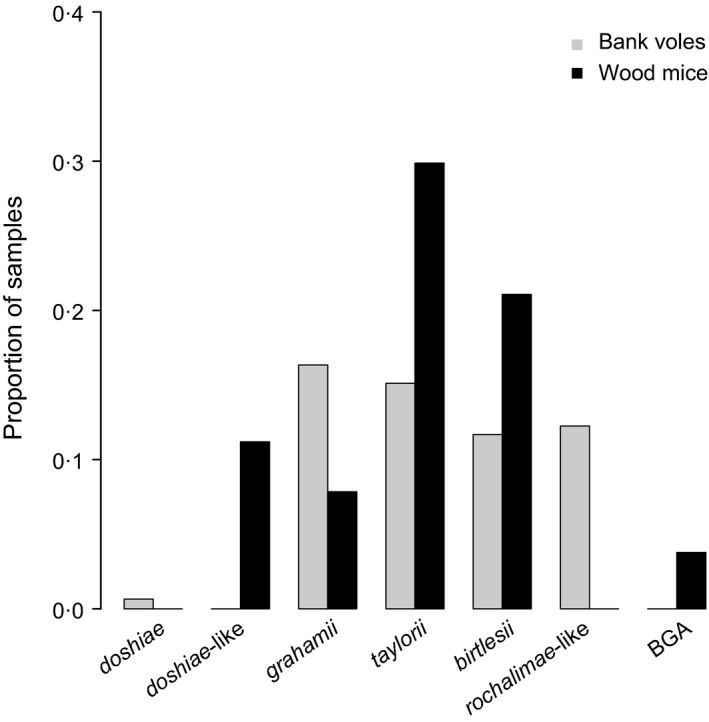
The proportion of blood samples that tested positive for infection with each *Bartonella* species in bank voles and wood mice across all sites. Infections were identified to species according to sequencing of the pITS region where possible, and according to the length of the pITS region in all other cases.

Amplicons of five broad size categories were obtained from the genus‐specific *Bartonella* PCR. Sequencing analyses confirmed that seven distinct species groups were represented, according to similarity to validated species in GenBank. Patterns of host associations were consistent across woodland sites (Fig. S2, Table S2); we therefore describe the combined data here. Three species (*B. grahamii*,* B. taylorii* and *B. birtlesii*) were found in both wood mice and bank voles (Fig. 1, Fig. S2). Two species (*B. rochalimae*‐like and *B. doshiae*) were found only in bank voles, and two species (BGA and *B. doshiae*‐like) were found only in wood mice (Fig. 1, Table S2).

### *Bartonella* in rodents: pITS variant‐level data

Sequences were obtained for 439 *Bartonella* pITS amplicons from wood mice (43·5% of pITS amplicons) and 391 amplicons from bank voles (56·6% of amplicons) (Table S2). Twenty‐six unique variants were identified (Table S2), including ten variants that were new to GenBank (see Table S4 for accession numbers). All variants shared at least 94% similarity (with the majority sharing 99–100% similarity) to their closest species match within GenBank, with their next closest species match sharing lower similarity (Table S11). We found no association between the proportion of pITS amplicons sequenced and the number of variants per *Bartonella* species found within each host species (Appendix S1.3; Fig. S1). We therefore assume that the host associations described below would not be affected by increased sequencing effort. Samples that were not sequenced were classified to species according to amplicon size only, and denoted as ‘unknown’ variant within that species group.

**Figure 2:**
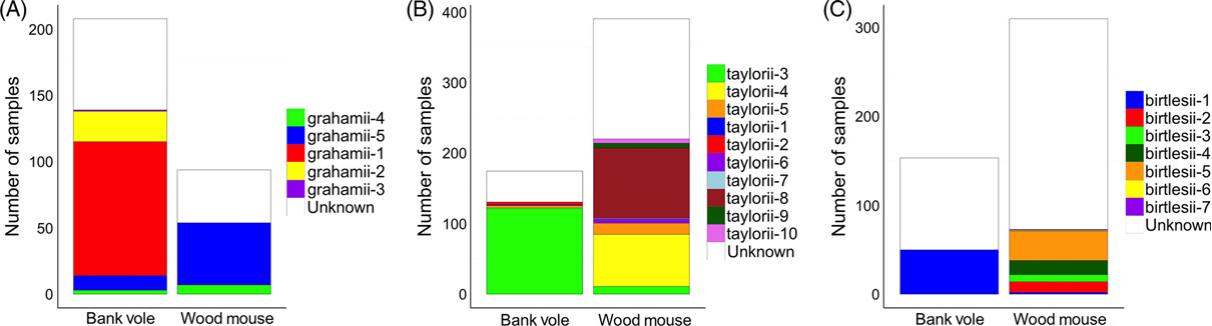
The number of each (A) *Bartonella grahamii* (B) *Bartonella taylorii* and (C) *Bartonella birtlesii* variant detected within wood mice and bank voles across all sites. Colour coding represents different variants within each *Bartonella* species group. Infections that were not sequenced are classed as ‘unknown’ variants (white). Classification of ‘unknown’ variants into their respective *Bartonella* species groups is based on pITS length.

Twenty‐two of the variants identified constituted three different *Bartonella* species groups and displayed varying degrees of host specificity. Five variants, each ~315 bp in length, shared highest percentage similarity with *B. grahamii* in GenBank (Table S11); three were bank vole specific (grahamii‐1, grahamii‐2 and grahamii‐3), and two were found in both host species (‘host‐shared’; grahamii‐4 and grahamii‐5), and while none were wood mouse specific, the majority of wood mouse infections comprised variants that were relatively rare in bank voles (Fig. 2A, Table S2). Ten variants, each ~350 bp in length, shared highest similarity with *B. taylorii* (Table S11); five were wood mouse specific (taylorii‐6, taylorii‐7, taylorii‐8, taylorii‐9 and taylorii‐10), and two were bank vole specific (taylorii‐1 and taylorii‐2; Fig. 2B, Table S2). The remaining three variants were host‐shared, although one was more common in bank voles (taylorii‐3) and two more common in wood mice (taylorii‐4 and taylorii‐5; Fig. 2B, Table S2). Finally, seven variants shared highest similarity with *B. birtlesii* (Table S11). Each was 370 bp in length, except for one, birtlesii‐4, which was 351 bp. The majority were wood mouse specific (birtlesii‐2, birtlesii‐3, birtlesii‐4, birtlesii‐5, birtlesii‐6 and birtlesii‐7), while one was host‐shared (birtlesii‐1) but far more common in bank voles (Fig. 2C, Table S2).

The four remaining variants each shared highest percentage similarity with a separate *Bartonella* species in GenBank. There were two variants with a pITS length of *c*. 290 bp. One matched most closely to *B. doshiae* (doshiae‐1, 292 bp), whereas the other (doshiae‐like‐1) was identical to variant ‘wbs011’ found in previous studies of rodent *Bartonella* in the UK (Table S11). This latter variant was classified as a *B. doshiae*‐like species (Telfer *et al*. 2005), owing to its high similarity to *B. doshiae* at the citrate synthase marker but divergence at the ITS region, and we retain that nomenclature here. Finally, there were two variants with a pITS length of ~460 bp. One (BGA‐1, 466 bp) was identical to a variant previously classified as a species called BGA (Telfer *et al*. 2007b), whereas the other (rochalimae‐like‐1, 461 bp) was identical to a sequence from a non‐isolated candidate species called *B. rudakovii* (Table S11). As this species is unconfirmed, we classify this variant as *B. rochalimae*‐like here, as candidatus *B. rudakovii* has been found to group closely with the species *B. rochalimae* according to similarity at the ITS region and at other markers (e.g. Diniz *et al*. 2009). Each of these four species groups was host specific: all amplicons of ~290 bp sequenced from bank voles (2 of 2) were identified as *B. doshiae*, while all those sequenced from wood mice (58 of 161) were *B. doshiae*‐like, and all amplicons of ~460 bp sequenced from bank voles (66 of 152) were identified as *B. rochalimae*‐like, while all of those sequenced from wood mice (35 of 55) were identified as BGA (Table S2).

### Comparison of *Bartonella* parasites found in wood mice and bank voles

**Figure 3:**
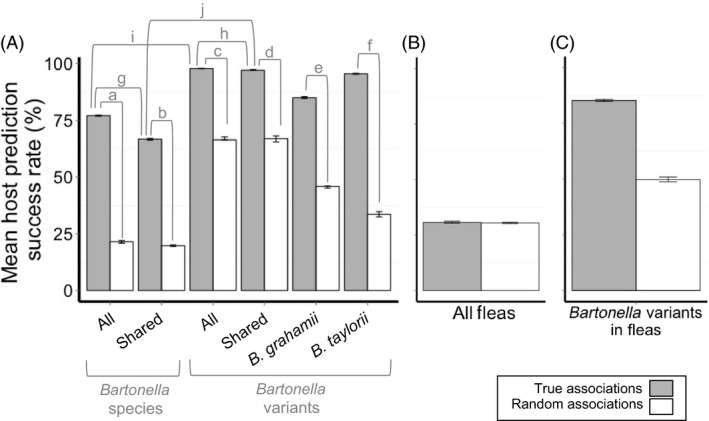
Mean percentage of individuals correctly identified to host species according to linear discriminant analyses where models were trained on (A) *Bartonella* infections of the hosts, (B) flea infestations of the hosts (χ^2^ = 0·02, *P *=* *0·88) and (C) *Bartonella* infections of the fleas infesting the hosts (χ^2^ = 28·7, *P *<* *0·001), using data from all three woodland sites combined. In each case, models were trained on random selections of 75% of host–parasite associations and used to predict the host identity of the remaining 25% of the data. This was done 1000 times in each case. Grey bars represent models trained on true host–parasite associations, while white bars represent models trained on random host–parasite associations. Differences between the predictive capabilities of each model were assessed using chi‐squared analyses. In Fig. (A), models were trained on host‐*Bartonella* infections identified either to species level (‘*Bartonella* species’) or to pITS variant level (‘*Bartonella* variants’), and ten comparisons were made, represented by the letters a–j. a: χ^2^ = 61·8, *P *<* *0·001, b: χ^2^ = 44·8, *P *<* *0·001, c: χ^2^ = 33·5, *P *<* *0·001, d: χ^2^ = 30·9, *P *<* *0·001, e: χ^2^ = 33·8, *P *<* *0·001, f: χ^2^ = 83·7, *P *<* *0·001, g: χ^2^ = 26·8, *P *<* *0·001, h: χ^2^ = 0·99, *P *=* *0·32, i: χ^2^ = 19·5, *P *<* *0·001, j: χ^2^ = 31·2, *P *<* *0·001. Linear discriminant analyses models could not be computed for *Bartonella birtlesii* variants alone as the distribution of the one variant shared between host species was highly skewed (birtlesii‐1, found only twice in wood mice but 50 times in bank voles; Table S2).

The assemblages of *Bartonella* detected in wood mice and bank voles were highly distinguishable according to the LDAs. Models trained on true host–parasite associations were consistently better at predicting host species than models trained on random associations (comparisons a–f Fig. 3A, Table S5). This was true whether *Bartonella* were identified to species level [Fig. 3A comparison ‘a’ (77·1% vs. 21·5%, χ^2^ = 61·8, *P *<* *0·001) and comparison ‘b’ (66·7% vs. 19·8%, χ^2^ = 44·8, *P *<* *0·001)] or to variant level [Fig. 3A comparison ‘c’ (97·8% vs. 66·4%, χ^2^ = 33·5, *P *<* *0·001) and comparison ‘d’ (97·1% vs. 66·9%, χ^2^ = 30·9, *P *<* *0·001)], and when considering associations of the variants within individual *Bartonella* species [Fig. 3A comparison ‘e’ (85·0% vs. 44·9%, χ^2^ = 33·8, *P *<* *0·001) and comparison ‘f’ (95·5% vs. 33·6%, χ^2^ = 83·7, *P *<* *0·001)]. However, the success of models trained on species‐level data was significantly reduced when the associations of the four host‐specific, single‐variant *Bartonella* species (*B. doshiae*,* B. doshiae*‐like, *B. rudakovii* and BGA) were omitted [Fig. 3A comparison ‘g’ (77·1% vs. 66·7%, χ^2^ = 26·8, *P *<* *0·001)]. In contrast, models trained on variant‐level data performed equally well whether incorporating all or just host‐shared *Bartonella* species [Fig. 3A comparison ‘h’ (97·8% vs. 97·1%, χ^2^ = 0·99, *P *=* *0·32)], and were always superior to models trained on species‐level data [Fig. 3A comparison ‘i’ (97·8% vs. 77·1%, χ^2^ = 19·5, *P *<* *0·001) and comparison ‘j’ (97·1% vs. 66·7%, χ^2^ = 31·2, *P *<* *0·001)].

### Rodent flea assemblages

**Figure 4:**
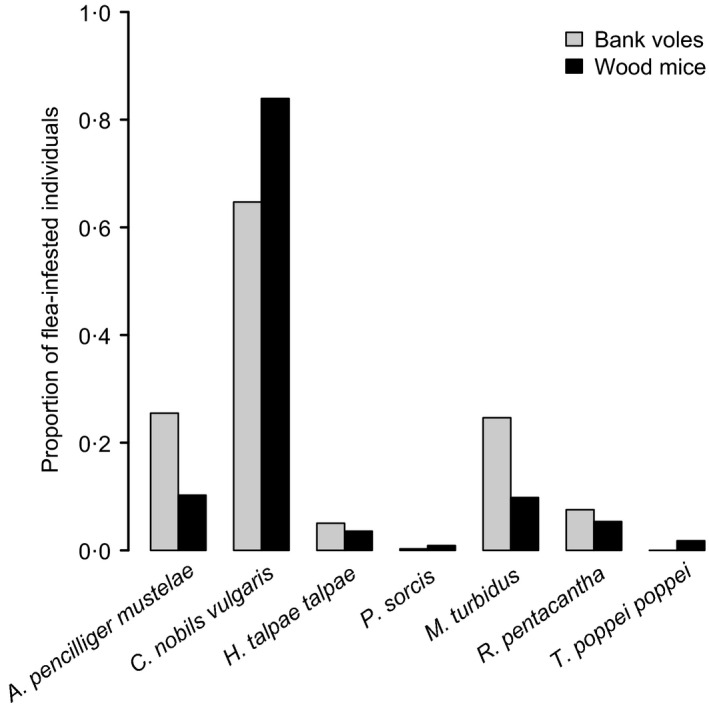
The proportion of flea‐infested wood mice and bank voles that were infested with at least one specimen of each species of flea detected in this study.

Fleas were collected from 224 wood mice (WM; 325 fleas) and 357 bank voles (BV; 589 fleas). Seven species were identified: *Amalareus penicilliger mustelae* (from 91 BV and 23 WM), *Ctenophthalmus nobilis vulgaris* (231 BV, 188 WM), *Hystrichopsylla talpae talpae* (18 BV, 8 WM), *Megabothris turbidus* (88 BV, 22 WM), *Palaeopsylla sorcis* (1 BV, 2 WM), *Rhadinopsylla pentacantha* (27 BV, 12 WM) and *Typhlocerus poppei poppei* (0 BV, 4 WM). All species of flea except *T. p. poppei* were found on both rodent species (Fig. 4). The assemblages of flea species collected from wood mice and bank voles were not distinguishable according to the LDA. Models trained on true host–flea associations were no better at predicting host species than models trained on random associations [Fig. 3B (30·4% mean prediction success vs. 30·7%, χ^2^ = 0·212, *P *=* *0·88), Table S7].

### *Bartonella* in rodent fleas

DNA was extracted from 881 fleas. *Bartonella* DNA was detected in 460 (52%) individual fleas, and in all flea species except *T. p. poppei*. pITS sequences were obtained for 382 *Bartonella* pITS amplicons, each from a separate flea. The remaining 78 *Bartonella*‐positive fleas were coinfected, and pITS amplicons were not sequenced. Thirty different variants were found (Table S8), representing eight *Bartonella* species (Table S11). Twenty variants matched those identified in rodent blood samples in this study; nine of which were wood mouse specific (doshiae‐like‐1, BGA‐1, taylorii‐6, taylorii‐7, taylorii‐8, taylorii‐9, taylorii‐10, birtlesii‐5 and birtlesii‐7), five were bank vole specific (doshiae‐1, rudakovii‐1, grahamii‐1, grahamii‐2 and taylorii‐2) and six were host‐shared (grahamii‐4, grahamii‐5, taylorii‐3, taylorii‐4, taylorii‐5 and birtlesii‐1) (Table S2). The remaining ten variants were novel to this study and to GenBank (they have now been added; Table S9). There were three *B. grahamii* variants (grahamii‐6, grahamii‐7 and grahamii‐8), two *B. taylorii* variants (taylorii‐11 and taylorii‐12), two *B. birtlesii* variants (birtlesii‐8 and birtlesii‐9) and one *B. doshiae* variant (doshiae‐2) (Table S11). One variant (tribocorum‐1) was most similar to *B. tribocorum*, a species previously found to infect rats (Heller *et al*. 1998), and never recorded from wood mice or bank voles in this study. One further variant (unknown‐1) did not closely match any known *Bartonella* species in GenBank (Table S11).

### Comparing *Bartonella* in fleas collected from wood mice and bank voles

A range of *Bartonella* pITS variants, including wood mouse specific, bank vole specific and host‐shared, were found in all flea species in which *Bartonella* DNA was detected (except *P. sorcis*, for which only a single *Bartonella* pITS amplicon was characterized; Table S8). However, the LDA showed that the species of rodent from which a *Bartonella*‐positive flea was collected was highly predictable based on the variant of *Bartonella* it was carrying [Fig. 3C (85·3% mean prediction success for models trained on true associations between flea *Bartonella* variants and rodent species vs. 49·8% for models trained on random associations, χ^2^ = 28·7, *P *<* *0·001), Table S10]. In other words, the assemblage of *Bartonella* variants found within fleas tended to reflect the assemblage of *Bartonella* variants found within the host species they were collected from. This pattern is unlikely to simply reflect recent acquisition of infections by fleas feeding on their current host, as the variants carried by fleas often did not match the variants carried by the rodent host from which they were collected (Table S13).

**Figure 5:**
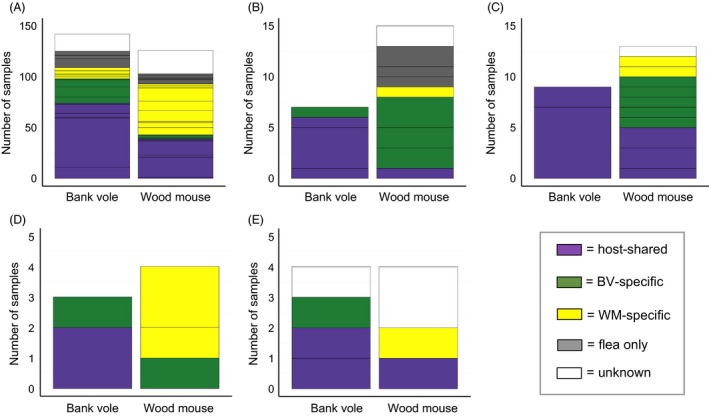
The number of (A) *Ctenophthalmus nobilis vulgaris* (B) *Megabothris turbidus* (C) *Amalareus penicilliger mustelae* (D) *Hystrichopsylla talpae talpae* and (E) *Rhadinopsylla pentacantha* taken from wood mice and bank voles that tested positive for *Bartonella* infection. Colour coding represents the host associations (according to this study) of the *Bartonella* pITS variants found within the fleas: purple = found in wood mice and bank voles, green = found only in bank voles, yellow = found only in wood mice, grey = found only in fleas. White represents *Bartonella* DNA in fleas that was not sequenced. Horizontal divisions within colour blocks represent multiple pITS variants within a host‐association category. The specific identities of variants identified in each flea species collected from each host species are shown in Table S8.

Host‐specific pITS variants were occasionally found in fleas collected from the alternative rodent species (Fig. 5). Wood mouse specific variants were found in *C. n. vulgaris* (doshiae‐like‐1, taylorii‐6, taylorii‐7, taylorii‐8, BGA‐1; Fig. 5A and Table S8) collected from bank voles, and bank vole specific variants were found in *C. n. vulgaris* (doshiae‐1, grahamii‐1, taylorii‐2; Fig. 5A and Table S8), *M. turbidus* (grahamii‐1, grahamii‐2, rudakovii‐1; Fig. 5B and Table S8), *A. p. mustelae* (grahamii‐1, grahamii‐2, rudakovii‐1; Fig. 5C and Table S8) and *H. t. talpae* (grahamii‐2; Fig. 5D and Table S8) collected from wood mice. No such pattern was found in *R. pentacantha* (Fig. 5E and Table S8) even though a similar number of pITS amplicons were sequenced for this flea species (*n* = 6) as for *H. t. talpae* (*n* = 7) for which evidence of between‐host species flea transfer was present. There was also no evidence of flea transfer for *P. sorcis*, but only a single specimen of this flea species was positive for *Bartonella* DNA. Examples of host‐specific variants in fleas collected from the alternative rodent species were also evident when considering only data from 2012 at MFG and RH, the site‐year combinations for which *Bartonella* sequences from both hosts and fleas were available (Fig. S3).

## Discussion

An ever‐expanding body of evidence clearly demonstrates that most parasite species infect multiple host species (Cleaveland, Laurenson & Taylor 2001; Taylor, Latham & Woolhouse 2001; Pedersen *et al*. 2005; Streicker, Fenton & Pedersen 2013). Where the same parasite endemically infects sympatric host species, between‐species transmission is assumed to be commonplace (i.e. a ‘true multihost parasite’; Fenton & Pedersen 2005), meaning a parasite reservoir potentially comprises an entire multihost community (Haydon *et al*. 2002) with transmission occurring somewhat freely between species. For medically important parasites, such a scenario would require potentially complex disease management across all host species (Fenton *et al*. 2015). In contrast to this conventional wisdom, however, we have shown that even with considerable overlaps in host species ecology, and despite the presence of host‐generalist vectors, the transmission of multihost parasites between endemically infected sympatric host species in the wild is surprisingly infrequent.

Overall, we found seven *Bartonella* species circulating within a host community of two sympatric rodent species, and three of these (*B. grahamii*,* B. taylorii* and *B. birtlesii*) infected both wood mice and bank voles. This is consistent with a previous study that used one of the same field sites (Manor Wood) (Telfer *et al*. 2007a). However, our genetic characterization of these parasites revealed considerable diversity within a partial ITS region of these three *Bartonella* species. Crucially, we found that each host species was associated with highly distinguishable assemblages of variants, and many of these variants were host specific. Furthermore, while some variants were shared across host species, these shared variants were always far more common in one host species than the other. Together, these results provide strong evidence for ‘covert host specificity’ among these variants, implying a general lack of parasite transmission between these two common sympatric rodent species, despite such transmission having previously been suggested from observed relationships between parasite prevalence and host densities (Telfer *et al*. 2007a).

We found clear evidence that most flea species are host‐generalists; in fact, all flea species except *T. p. poppei* were found on both wood mice and bank voles, and overall, the assemblages of fleas associated with each host species were indistinguishable according to our linear discriminant analyses. However, the dispersal of these generalist vectors between host species appeared to be limited, which may restrict opportunities for between‐species *Bartonella* transmission. We identified the genetic variants of *Bartonella* being carried by fleas and found that overall, the identity of the host species from which a flea was taken could be determined by looking only at the *Bartonella* variant carried by that flea. The assemblage of *Bartonella* variants found within the flea community therefore has clear structure, which is strongly correlated with the rodent host species that fleas were collected from. This suggests that separate communities of the same flea species may circulate largely independently within each host species population and that transfer of individual fleas between these discrete pools is rare. This seems reasonable, as the flea species found at our study sites are mostly nest‐dwellers that feed opportunistically on hosts entering their nests (Marshall 1981; Krasnov 2008). Flea movement between species is therefore likely to require close mouse–vole contact, or use of the same habitat space by different host individuals for a sufficient period of time (Krasnov & Khokhlova 2001), which may be infrequent due to differences in activity patterns and microhabitat usage by wood mice and bank voles (Watts 1968; Crawley 1969; Greenwood 1978; Canova 1993). Indeed, wood mice and bank voles were only occasionally captured at the same trap location during a given monthly session across our study sites (median proportion of multispecies trap locations per session was 0·2 across all sessions and sampling sites; Table S12), indicating some differentiation in microhabitat use within the same broad woodland area.

As a consequence of limited between‐species vector dispersal, opportunities for between‐species parasite transmission may be rare (i.e. an encounter barrier), even when host species are infected by the same vector species. This potentially counters the complex view of parasite persistence and control within multispecies reservoirs (Haydon *et al*. 2002). However, if host‐specific variants are physiologically capable of infecting a wider range of host species given the opportunity, between‐species transmission may occur if barriers to encounter break down, for example due to anthropogenic shifts in community structure (i.e. a ‘potential multihost parasite’ becoming a ‘true multihost parasite’; Fenton & Pedersen 2005). Here, however, we found evidence that at least some host‐specific *Bartonella* variants were unable to infect the other species, possibly due to physiological incompatibility, as some fleas were found carrying these host‐specific variants on the other, uninfected host species. In fact, as we did not sequence *Bartonella* DNA from any coinfected fleas, it is possible that we underestimate the occurrence of between‐species flea transfer here, as coinfected fleas may arise as a result of feeding sequentially on multiple host individuals, and possibly different host species, infected with different pathogens. Such compatibility barriers have been found in Irish rodent communities, where wood mice were endemically infected with *Bartonella* but sympatric bank voles were not, despite harbouring *Bartonella*‐positive fleas (Telfer *et al*. 2005). Laboratory inoculation experiments have also shown that *Bartonella* infections often only establish in species of wild rodents when challenged with a variant originally obtained from that same species (Kosoy *et al*. 2000). It therefore seems likely that should ecological barriers to between‐host vector transfer break down in the future (e.g. due to environmentally driven changes to host or vector movement), initial incompatibility barriers may prevent or slow the emergence of regular between‐species *Bartonella* transmission, until new variants able to infect multiple host species evolve and increase in frequency (Antia *et al*. 2003; Lloyd‐Smith *et al*. 2009).

Interestingly, we found six shared variants of *Bartonella*, all of which were far more common in one host species than the other. Given the occasional occurrence of fleas carrying variants never found in the host species from which they were collected, it seems that between‐species flea transfer does occur, at a rate which is sufficient for those few shared variants to maintain a relatively constant, but low, degree of host generalism (indicative of spillover dynamics; Fenton & Pedersen 2005). Alternatively, it may be that host generalism is a more dynamic phenomenon and that our data represent a snapshot in evolutionary time such that we are witnessing the evolution of these variants from host‐specialists to host‐generalists (or vice versa). It would therefore be fascinating to conduct a longer‐term study of this system to see the extent to which variants change in frequency in the two host species over time, and therefore whether between‐species transmission is becoming more or less common.

Our findings provide compelling evidence that the ecology of host‐generalist vectors could inhibit between‐species parasite transmission. We acknowledge that our conclusions about between‐species vector movement are drawn from proxy evidence of associations between individual fleas and host species, and an investigation of the genetic structure of the flea populations may help to assess the frequency with which individual fleas transfer between sympatric species and promote between‐species transmission. The generality of our findings will also depend on parasite transmission mode and the off‐host dispersal capabilities of other vector types (Randolph 1998). For example, vectors that engage in frequent host‐independent dispersal (e.g. dipterans such as mosquitoes) have the opportunity to feed sequentially on different host species more often and thus are less likely to represent a barrier to the between‐species transmission of multihost parasites. Furthermore, parasites transmitted by direct contact may have fewer opportunities to cross between host species. For example, it was previously shown that risk of infection with the directly transmitted cowpox virus is not influenced by between‐species transmission for sympatric populations of wood mice and bank voles (Begon *et al*. 1999; Carslake *et al*. 2006), presumably due to infrequent appropriate interspecies encounters. In contrast, opportunities for between‐species exposure for parasites with environmental transmission stages (e.g. intestinal helminths) may be more frequent, with different vector‐borne parasites lying at different points along a continuum between these two extremes. Identifying general trends in the occurrence of between‐species transmission based on broad host and parasite ecology would improve our understanding of disease transmission within complex ecological communities.

In conclusion, our results show that the transmission of multihost parasites between sympatric host species is not inevitable, and cannot necessarily be predicted based on shared host ecologies alone, nor on the presence of host‐generalist vectors. We emphasise that, in fact, between‐species transmission may be a lot more rare than previously assumed. Thus, separate populations of the same parasite species may often circulate and persist independently in different sympatric host species populations. This challenges conventional wisdom surrounding the control of multihost parasites and, if a general phenomenon, suggests that control interventions would likely need to be multipronged, aiming to reduce infection independently in multiple host species.

## Data accessibility

All data associated with this study have been deposited in the Dryad Digital Repository: http://dx.doi.org/10.5061/dryad.gm061 (Withenshaw *et al*. 2016). As data analyses are ongoing, release of data has been embargoed for 1 year from the date of publication. GenBank accession numbers of all sequences included in this paper, including those identified for the first time here, are shown in Tables S4, S9 and S11.

## Supporting information


**Appendix S1.** Additional methodological details.

**Fig. S1.** Relationship between the proportions of positive samples per *Bartonella* species that were sequenced and the number of *Bartonella* variants detected.

**Fig. S2.** Proportion of blood samples testing positive for infection with each *Bartonella* species at each field site.

**Fig. S3.** Number of *Bartonella*‐positive fleas of each species taken from wood mice and bank voles at MFG and RH during 2012.

**Table S1.** Number of individual wood mice and bank voles captured and number of blood samples collected from each rodent species at each field site.

**Table S2.** The 26 *Bartonella* partial 16S‐23S ITS sequence variants detected in this study and where they were found.

**Table S3.** As Table S2, but only samples from animals not exposed to treatment are presented.

**Table S4.** GenBank accession numbers of the ten novel Bartonella partial 16S‐23S ITS sequence variants detected in rodent blood samples in this study.

**Table S5.** Results of linear discriminant analyses that modelled host species identity based on either the species‐level (S) or variant‐level (V) identification of *Bartonella* parasites with which they were infected.

**Table S6.** As Table S5, but using a reduced data set that includes only a single record of a particular *Bartonella* species or pITS variant for each individual.

**Table S7.** Results of linear discriminant analyses that modelled host species identity based on the morphological identification of the flea species collected from them.

**Table S8.** The species identity and *Bartonella* infection status of fleas collected from rodents.

**Table S9.** GenBank accession numbers of the ten *Bartonella* partial 16S‐23S ITS sequence variants detected in fleas only.

**Table S10.** Results of linear discriminant analyses that modelled host species identity based on the variant of *Bartonella* carried by fleas collected from them.

**Table S11.** *Bartonella* species submissions in GenBank with which each pITS variant in this study shares highest and second highest similarity.

**Table S12.** Proportions of trap locations at which both wood mice and bank voles were captured during each trapping session at each site.

**Table S13.** Comparison of the *Bartonella* pITS variants found in individual fleas and the variants found in the rodent hosts from which each flea was collected.
