# Supplementary material for: Multihost Bartonella parasites display covert host specificity even when transmitted by generalist vectors
Source: J Anim Ecol. 2016 Aug 16;85(6):1442–52. doi: 10.1111/1365-2656.12568 (PMC5082552; doi:10.1111/1365-2656.12568)
Supplement: Supplementary file 1 — Appendix S1. Additional methodological details. [file JANE-85-1442-s001.pdf]

Appendix S1 Additional methodological details

1. Field methods

Sherman live-traps (Alana Ecology, UK; dimensions 8.9cm x 7.6cm x 22.9cm) were set within discrete trapping grids at each site. Trapping stations within each grid were 10m apart, and two traps were placed at each station. There were four grids at MW; two measured 50m x 50m (72 traps each) and two measured 70m x 70m (128 traps each), totalling 400 traps. There were three grids at both MFG and RH, all measuring 50m x 50m (72 traps each), totalling 216 traps at each site. Traps were set for three consecutive nights every four weeks from May to December in 2011 at MW, and in 2012 at MFG and RH, totalling eight monthly trapping sessions per year. Traps were checked for animals each morning.

2. DNA extraction and pITS variant-level identification of *Bartonella* infections

DNA was extracted from blood samples using DNAzol BD reagent© (Invitrogen™). Extraction success was confirmed by quantification of DNA using the Qubit™ double-stranded DNA high-sensitivity assay (Invitrogen, UK). Extracts were used as templates in a genus-specific PCR targeting a 300-500bp fragment of the *Bartonella* 16S-23S internal transcribed spacer region (Telfer *et al*. 2005) (hereafter referred to as the partial ITS region, pITS).

Some species of *Bartonella* can be differentiated by length polymorphism of the pITS region (Roux & Raoult 1995; Birtles *et al*. 2000; Houpikian & Raoult 2001; Telfer *et al*. 2005), but this method cannot differentiate between *Bartonella* species that have similar pITS length nor can it identify genetic variation within *Bartonella* species. A random subset of amplicons from each pool of putative *Bartonella* species from each host species and site were therefore sequenced in the forward and reverse directions using an Applied Biosystems ABI PRISM® 3130xl Genetic Analyzer. Samples from single infections (i.e. not coinfections) were prioritised for sequencing but where few single infections existed for a particular *Bartonella*-host-site combination, amplicons from coinfections were sequenced by first extracting and purifying PCR products from an agarose gel using a MinElute® Gel Extraction Kit (Qiagen, UK). Sequence quality in all cases was assessed by visual inspection of chromatograms in Geneious Pro v5.6.6, and consensus sequences were constructed. Unique pITS sequence variants were identified and compared by alignment using the default settings of ClustalW within Geneious Pro. All unique variants were also compared to known *Bartonella* species within the NCBI nucleotide database using BLAST searches and grouped into species according to their closest match (the sequence of a named *Bartonella* species with which it shared highest percentage identity).

3. Assessing potential bias in the sampling of *Bartonella* variants from different host species and sites:

An unequal proportion of positive infections were characterised at the sequence level across *Bartonella* species, host species and sites. Before comparing the parasite assemblages found in each host species, we therefore assessed whether this sampling bias may have affected the abundance of variants detected in each case. The number of variants per *Bartonella* species per host species per site was used as a response variable in a generalized linear model, with log link for quasi-Poisson errors (Streicker *et al*. 2010), and the following explanatory variables were investigated: the proportion of positive samples that were characterised at the sequence level for each *Bartonella*-host-site combination (i.e. sequencing effort), and 2-way interactions between sequencing effort,

host species and *Bartonella* species. These interaction terms were included to control for the fact that the rate at which novel variants are detected may vary across host and/or *Bartonella* species.

There was no association between the proportion of positive samples sequenced and the variant richness detected per *Bartonella* species-host-site combination (GLM: Proportion sequenced $F_{1,26}$ = 0.02, p = 0.90; Fig. S1), and this result was consistent across host species (GLM: Proportion sequenced*Host species $F_{1,26}$ = 0.08, p = 0.78) and *Bartonella* species (GLM: Proportion sequenced**Bartonella* species $F_{5,26}$ = 0.40, p = 0.84). Further sequencing of the positive samples collected is therefore unlikely to increase the number of *Bartonella* spp. variants found in each host species at each site, and so the assemblages detected would appear to be representative of the true patterns in the community.

4. DNA extraction from fleas

DNA was extracted from individual fleas using a Promega Wizard® Genomic DNA Purification Kit (Promega Corporation, USA). 1μL of each extract was used in a PCR targeting the conserved invertebrate 18S rRNA gene (primers '1' and '6' of Hendriks *et al.* 1991) to confirm DNA extraction success. Presence of *Bartonella* in individual fleas was determined using the genus-specific PCR assay described above. pITS amplicons were sequenced from all positive, non-coinfected fleas.

References

Telfer, S., Bown, K., Sekules, R. & Begon, M. (2005). Disruption of a host-parasite system following the introduction of an exotic host species. *Parasitology*, 130, 661-668.

Roux, V. & Raoult, D. (1995). Inter-species and intra-species identification of *Bartonella* (*Rochalimaea*) species. *J Clin Microbiol*, 33, 1573–1579.

Birtles, R., Hazel, S., Bown, K., Raoult, D., Begon, M. & Bennett, M. (2000). Subtyping of uncultured bartonellae using sequence comparison of 16 S/23 S rRNA intergenic spacer regions amplified directly from infected blood. *Mol Cell Probes*, 14, 79-87.

Houpikian, P. & Raoult, D. (2001). 16S/23S rRNA intergenic spacer regions for phylogenetic analysis, identification, and subtyping of *Bartonella* species. *J Clin Microbiol*, 39, 2768–2778.

Streicker, D.G., Turmelle, A. S., Vonhof, M. J., Kuzmin, I. V., McCracken, G. F. & Rupprecht, C. E. (2010). Host phylogeny constrains cross-species emergence and establishment of Rabies virus in bats. *Science*, 329, 676–679.

Hendriks, L., de Baere, R., Van de Peer, Y., Neefs, J., Goris, A. & de Wachter, R. (1991). The evolutionary position of the Rhodophyte *Porphyra umbilicalis* and the Basidiomycete *Leucosporidium scottii* among other eukaryotes as deduced from complete sequences of small ribosomal subunit RNA. *J Mol Evol*, 32, 167–177.
