# Supplementary material for: Multihost Bartonella parasites display covert host specificity even when transmitted by generalist vectors
Source: J Anim Ecol. 2016 Aug 16;85(6):1442–52. doi: 10.1111/1365-2656.12568 (PMC5082552; doi:10.1111/1365-2656.12568)

Figure S1 Relationship between the proportion of positive samples per Bartonella species that were sequenced per host species per site, and the number of variants for that Bartonella species that were detected. Symbols are colour-coded by Bartonella species, with different symbol shapes for host species. A GLM revealed no significant association overall or within each host species or Bartonella species subset (see Appendix S1).

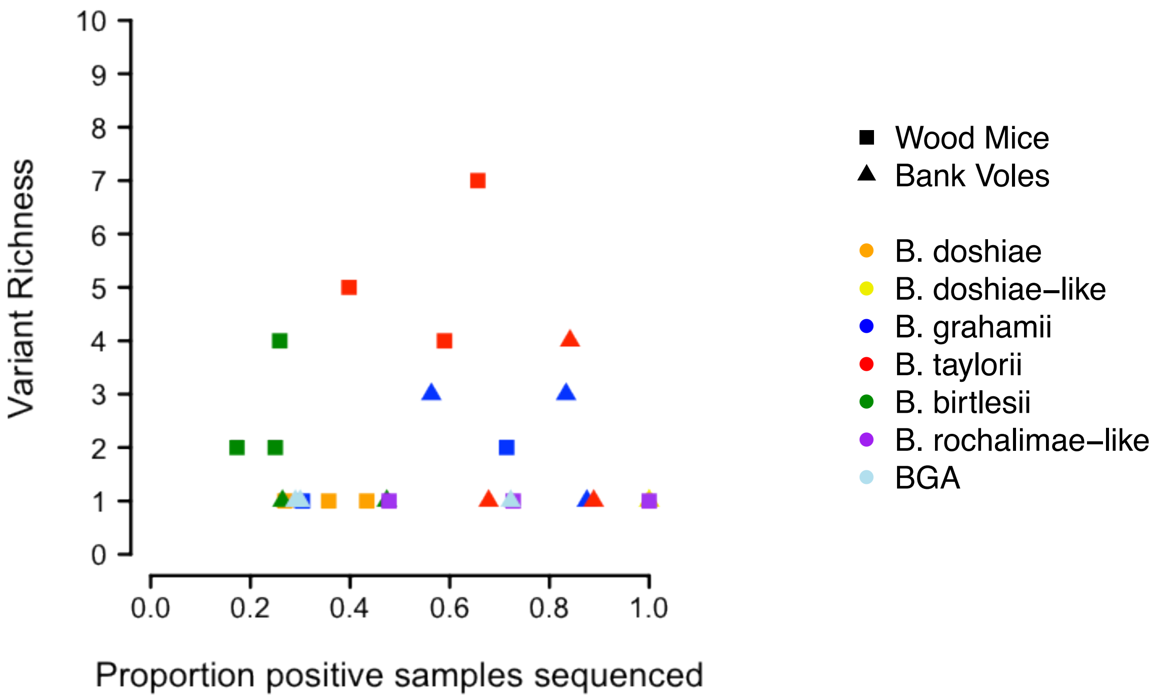

Supplement: Supplementary file 2 — Fig. S1. Relationship between the proportions of positive samples per Bartonella species that were sequenced and the number of Bartonella variants detected. [file JANE-85-1442-s002.pdf]
