# Supplementary material for: Multihost Bartonella parasites display covert host specificity even when transmitted by generalist vectors
Source: J Anim Ecol. 2016 Aug 16;85(6):1442–52. doi: 10.1111/1365-2656.12568 (PMC5082552; doi:10.1111/1365-2656.12568)

Figure S2 The proportion of blood samples that tested positive for infection with each Bartonella species in (a) Manor Wood (b) Maresfield & Gordale and (c) Rode Hall. Infections were identified to species according to sequencing of the pITS region where possible, and according to the length of the pITS region in all other cases. Grey bars = Bank Voles, Black bars = Wood Mice.

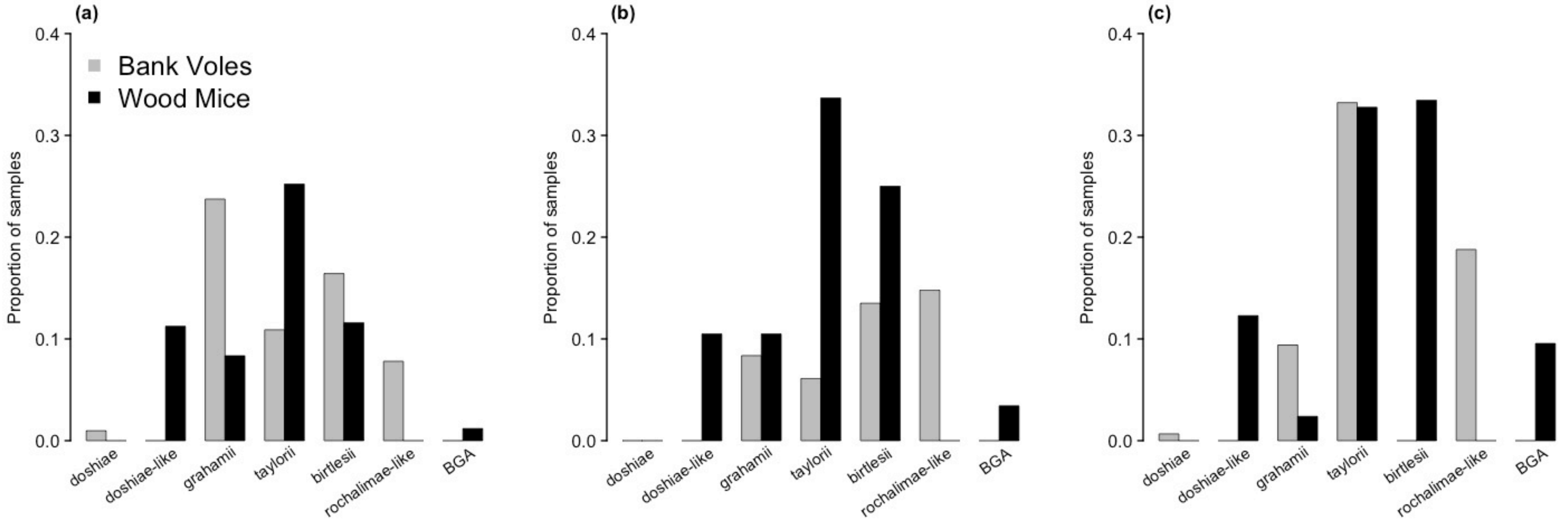

Supplement: Supplementary file 3 — Fig. S2. Proportion of blood samples testing positive for infection with each Bartonella species at each field site. [file JANE-85-1442-s003.pdf]
