# Supplementary material for: Multihost Bartonella parasites display covert host specificity even when transmitted by generalist vectors
Source: J Anim Ecol. 2016 Aug 16;85(6):1442–52. doi: 10.1111/1365-2656.12568 (PMC5082552; doi:10.1111/1365-2656.12568)

Figure S3 The number of (a) *C. n. vulgaris* (b) *M. turbidus* (c) *A. p. mustelae* (d) *H. t. talpae* and (e) *R. pentacantha* taken from wood mice and bank voles that tested positive for *Bartonella* infection. Colour-coding represents the host associations (according to this study) of the *Bartonella* pITS variants found within the fleas: purple = found in wood mice and bank voles, green = found only in bank voles, yellow = found only in wood mice, grey = found only in fleas. White represents infections in fleas that were not sequenced. Horizontal divisions within colour blocks represent multiple pITS variants within a host-association category. The specific identities of variants identified in each flea species collected from each host species are shown in Table S8.**Data are from 2012 only, and from MFG and RH only.**

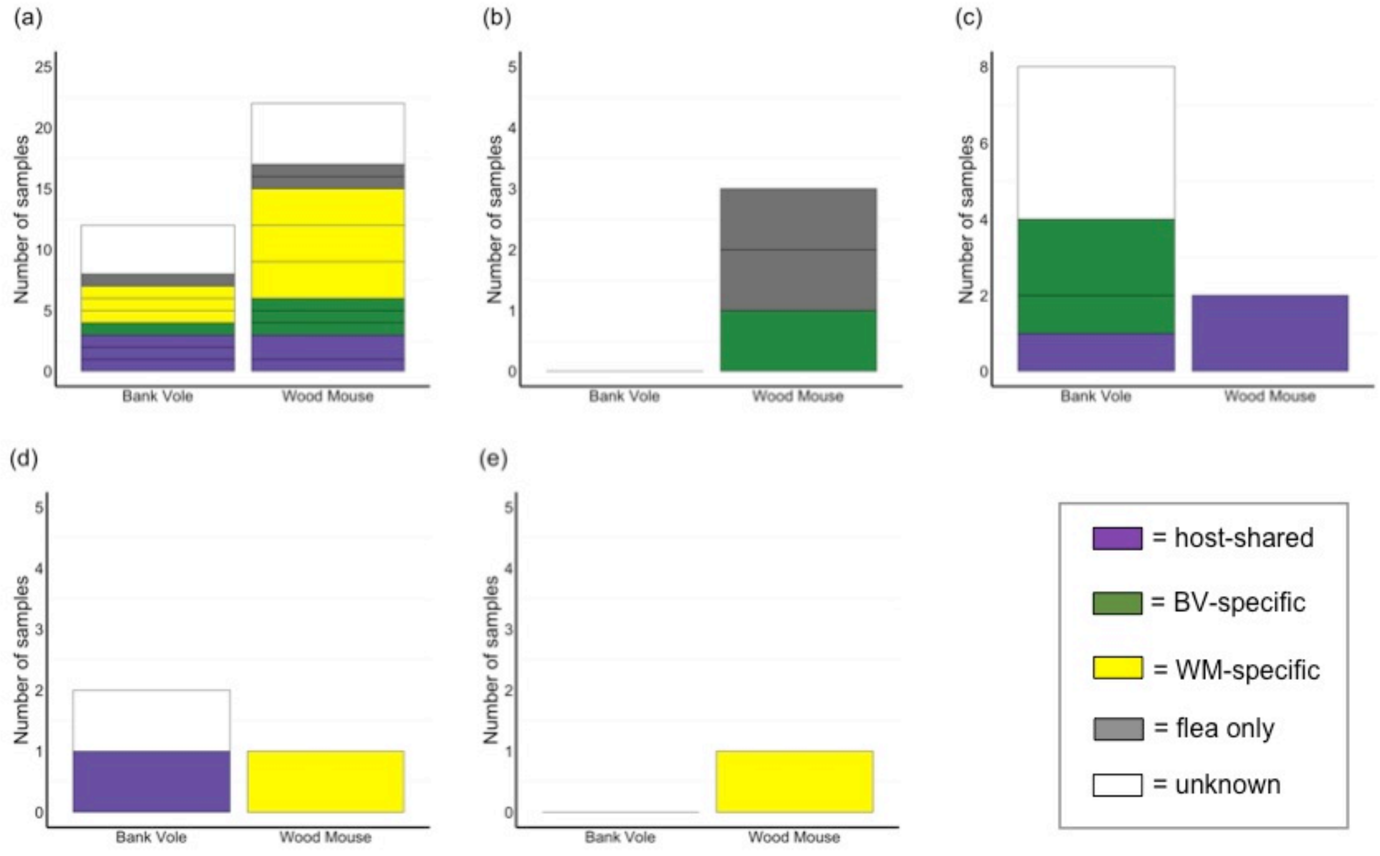

Supplement: Supplementary file 4 — Fig. S3. Number of Bartonella‐positive fleas of each species taken from wood mice and bank voles at MFG and RH during 2012. [file JANE-85-1442-s004.pdf]
