# Supplementary material for: Multihost Bartonella parasites display covert host specificity even when transmitted by generalist vectors
Source: J Anim Ecol. 2016 Aug 16;85(6):1442–52. doi: 10.1111/1365-2656.12568 (PMC5082552; doi:10.1111/1365-2656.12568)
Supplement: Supplementary file 5 — Table S1. Number of individual wood mice and bank voles captured and number of blood samples collected from each rodent species at each field site. [file JANE-85-1442-s005.pdf]

Table S1 The number of individual wood mice and bank voles captured and the number of blood samples collected from each species at each field site.

		Manor Wood	Maresfield & Gordale	Rode Hall
Wood mice	# blood samples	587	496	293
	# individuals	303	168	272
Bank voles	# blood samples	615	311	298
	# individuals	377	190	184
