# Supplementary material for: Multihost Bartonella parasites display covert host specificity even when transmitted by generalist vectors
Source: J Anim Ecol. 2016 Aug 16;85(6):1442–52. doi: 10.1111/1365-2656.12568 (PMC5082552; doi:10.1111/1365-2656.12568)
Supplement: Supplementary file 6 — Table S2. The 26 Bartonella partial 16S‐23S ITS sequence variants detected in this study and where they were found. [file JANE-85-1442-s006.pdf]

Table S2 The twenty-six *Bartonella* partial 16S-23S ITS sequence variants detected in this study. Sequence variants are grouped into *Bartonella* species groups based on their closest match to known *Bartonella* species within GenBank. Number of isolates of each pITS variant found in wood mice and bank voles at each woodland site are given, along with the number of "unknown" sequence variants of each putative *Bartonella* species. Variants in green were only found in bank voles; Variants in yellow were only found in wood mice; Variants in purple were found in both rodent species.

***Bartonella* species**	**pITS variant**	**Number of occurrences**							
		**All sites**		**MW**		**MFG**		**RH**	
		**WM**	**BV**	**WM**	**BV**	**WM**	**BV**	**WM**	**BV**
B. doshiae	*Unknown*		*0*		*0*				*0*
	doshiae-1		2		1				1
***B. doshiae*-like**	*Unknown*	*103*		*49*		*31*		*23*	
	doshiae-like-1	58		20		23		15	
***B. rochalimae*-like**	*Unknown*		*84*		*11*		*31*		*42*
	rochalimae-like-1		68		37		15		16
**BGA**	*Unknown*	*20*		*1*		*6*		*13*	
	BGA-1	35		7		11		17	
B. birtlesii	*Unknown*	*237*	*103*	*58*	*77*	*100*	*26*	*79*	
	birtlesii-1	2	50		30	1	20	1	
	birtlesii-2	12				12			
	birtlesii-3	8				8			
	birtlesii-4	16		16					
	birtlesii-5	33		6		9		18	
	birtlesii-6	1		1					
	birtlesii-7	1		1					
B. grahamii	*Unknown*	*40*	*69*	*23*	*59*	*16*	*4*	*1*	*6*
	grahamii-1		101		83		18		
	grahamii-2		23						23
	grahamii-3		1		1				
	grahamii-4	7	3			7	3		
	grahamii-5	47	12	18	7	25	5	4	
B. taylorii	*Unknown*	*171*	*44*	*77*	*21*	*53*	*2*	*41*	*21*
	taylorii-1		1						1
	taylorii-2		4						4
	taylorii-3	11	122	6	42	3	8	2	72
	taylorii-4	74	1	26		30	1	18	
	taylorii-5	16	3			15		1	3
	taylorii-6	6				6			
	taylorii-7	1				1			
	taylorii-8	98		19		47		32	
	taylorii-9	7		4		3			
	taylorii-10	6		1		5			
