# Supplementary material for: Multihost Bartonella parasites display covert host specificity even when transmitted by generalist vectors
Source: J Anim Ecol. 2016 Aug 16;85(6):1442–52. doi: 10.1111/1365-2656.12568 (PMC5082552; doi:10.1111/1365-2656.12568)
Supplement: Supplementary file 7 — Table S3. As Table S2, but only samples from animals not exposed to treatment are presented. [file JANE-85-1442-s007.pdf]

Table S3 As Table S2 but only samples from animals not exposed to treatment are presented (i.e. those taken from animals on observation grids and from treatment grids prior to the onset of treatment). All associations between variants and host species are the same as when considering all samples, except for Type 11, which was now only found in wood mouse samples even though it was found in both host species when considering all samples. No variants that were classed as host-specific when considering all samples were classed as host-shared when considering only samples from animals not exposed to treatment. Two variants were not found at all in this restricted set of samples (*B. birtlesii* variants birtlesii-6 and birtlesii-7).

***Bartonella* species**	**pITS variant**	**Number of occurrences**							
		**All sites**		**MW**		**MFG**		**RH**	
		**WM**	**BV**	**WM**	**BV**	**WM**	**BV**	**WM**	**BV**
B. doshiae	*Unknown*		*0*		*0*				*0*
	doshiae-1		2		1				1
***B. doshiae*-like**	*Unknown*	*103*		*49*		*31*		*23*	
	doshiae-like-1	40		20		13		7	
***B. rochalimae*-like**	*Unknown*		*84*		*11*		*31*		*42*
	rochalimae-like-1		44		26		9		9
**BGA**	*Unknown*	*20*		*1*		*6*		*13*	
	BGA-1	23		3		7		13	
B. birtlesii	*Unknown*	*237*	*103*	*58*	*77*	*100*	*26*	*79*	
	birtlesii-1	2	41		23	1	18	1	
	birtlesii-2	8				8			
	birtlesii-3	6				6			
	birtlesii-4	5		5					
	birtlesii-5	24		4		7		13	
	birtlesii-6								
	birtlesii-7								
B. grahamii	*Unknown*	*40*	*69*	*23*	*59*	*16*	*4*	*1*	*6*
	grahamii-1		77		64		13		
	grahamii-2		14						14
	grahamii-3		1		1				
	grahamii-4	2	3			2	3		
	grahamii-5	20	6	7	2	13	4		
B. taylorii	*Unknown*	*171*	*44*	*77*	*21*	*53*	*2*	*41*	*21*
	taylorii-1		1						1
	taylorii-2		3						3
	taylorii-3	8	94	4	40	3	8	1	46
	taylorii-4	39		15		15		9	
	taylorii-5	10	3			9		1	3
	taylorii-6	4				4			
	taylorii-7	1				1			
	taylorii-8	61		12		27		22	
	taylorii-9	3		3					
	taylorii-10	5		1		4			
