# Supplementary material for: Multihost Bartonella parasites display covert host specificity even when transmitted by generalist vectors
Source: J Anim Ecol. 2016 Aug 16;85(6):1442–52. doi: 10.1111/1365-2656.12568 (PMC5082552; doi:10.1111/1365-2656.12568)
Supplement: Supplementary file 8 — Table S4. GenBank accession numbers of the ten novel Bartonella partial 16S‐23S ITS sequence variants detected in rodent blood samples in this study. [file JANE-85-1442-s008.pdf]

Table S4 The GenBank accession numbers of the ten novel *Bartonella* partial 16S-23S ITS sequence types detected in rodent blood samples in this study. Sequence types are grouped into *Bartonella* species groups based on their closest match to existing named *Bartonella* species within GenBank.

***Bartonella* species**	**pITS variant**	**BLAST accession number**
B. doshiae	doshiae-1	KU589237
B. grahamii	grahamii-2	KU589238
	grahamii-3	KU589239
B. taylorii	taylorii-1	KU589240
	taylorii-2	KU589241
	taylorii-6	KU589242
	taylorii-10	KU589243
B. birtlesii	birtlesii-2	KU589244
	birtlesii-6	KU589245
	birtlesii-7	KU589246
