# Supplementary material for: Multihost Bartonella parasites display covert host specificity even when transmitted by generalist vectors
Source: J Anim Ecol. 2016 Aug 16;85(6):1442–52. doi: 10.1111/1365-2656.12568 (PMC5082552; doi:10.1111/1365-2656.12568)
Supplement: Supplementary file 9 — Table S5. Results of linear discriminant analyses that modelled host species identity based on either the species‐level (S) or variant‐level (V) identification of Bartonella parasites with which they were infected. [file JANE-85-1442-s009.pdf]

Table S5 Results of linear discriminant analyses that modelled host species identity based on either the species-level (S) or variant-level (V) identification of *Bartonella* parasites with which they were infected. Percentage successful prediction of host species is given for each model, where the model was first trained using 75% of real host-parasite associations (75:25 real) and then using 75% of randomised host-parasite associations (75:25 random). Mean percentage success of 1000 iterations is given, with 95% confidence intervals in brackets, and the success rates of models trained on the real data versus randomised data were compared using a Chi squared analysis. An LDA was performed according to infections with all seven *Bartonella* species (species-level and variant-level) and then according to infections with host-shared *Bartonella* species only (i.e. *B. grahamii*, *B. taylorii* and *B. birtlesii*) (species-level and variant-level). LDAs were also performed using infection data for each of the three host-shared species independently (variant-level only), as it is possible that covert host-specificity exists for some *Bartonella* parasites but not others. Analyses were performed using data from all sites combined, and from each woodland site separately (MW = Manor Wood, MFG = Maresfield & Gordale, RH = Rode Hall). Sample sizes (n) are given in each case. *Bartonella* species or variants observed on <5 occasions were omitted, as inclusion of very rare species/variants introduced computational problems when performing model validation. Infections with *B. doshiae* were not included in any analyses as the number of infections observed did not exceed five at any of the sites or when data from all sites were combined. LDA models could not be computed using infections with variants of *B. birtlesii* alone at any site, or variants of *B. grahamii* or *B. taylorii* alone at RH as once rare variants had been discounted, the remaining variants were largely host-specific.

Data used	Test	% Prediction success			
		**All Sites**	**MW**	**MFG**	**RH**
All *Bartonella* spp. (S)<br>n = 1419 all sites<br>n = 595 MW<br>n = 458 MFG<br>n = 366 RH	75:25 real	77.1 (76.8 – 77.3)	88.7 (88.3 – 89.1)	67.2 (66.5 – 67.9)	70.5 (70.1 – 70.9)
	75:25 random	21.5 (20.9 – 22.1)	59.1 (57.5 – 60.8)	47.3 (46.3 – 48.3)	46.7 (45.1 – 48.3)
	χ^2	61.8, $p < 0.001$	22.7, $p < 0.001$	8.09, $p = 0.004$	11.7, $p < 0.001$
Shared *Bartonella* spp. (S)<br>n = 1201 all sites<br>n = 520 MW<br>n = 383 MFG<br>n = 298 RH	75:25 real	66.7 (66.3 – 67.1)	83.9 (83.4 – 84.4)	59.1 (58.3 – 59.9)	51.8 (51.3 – 52.4)
	75:25 random	19.8 (19.4 – 20.2)	42.4 (40.3 – 44.5)	56.3 (55.9 – 56.8)	29.4 (28.0 – 30.7)
	χ^2	44.8, $p < 0.001$	37.0, $p < 0.001$	0.16, $p = 0.69$	10.4, $p = 0.001$
All *Bartonella* spp. (V)<br>n = 820 all sites<br>n = 318 MW<br>n = 267 MFG<br>n = 213 RH	75:25 real	97.8 (97.7 – 97.9)	96.9 (96.7 – 97.0)	95.0 (94.7 – 95.2)	96.2 (95.6 – 96.8)
	75:25 random	66.4 (65.2 – 67.7)	47.4 (46.2 – 48.7)	57.4 (56.5 – 58.3)	58.2 (56.6 – 60.0)
	χ^2	33.5, $p < 0.001$	60.2, $p < 0.001$	39.0, $p < 0.001$	$\chi^2 = 41.0$, $p < 0.001$
Shared *Bartonella* spp. (V)<br>n = 659 all sites<br>n = 254 MW<br>n = 218 MFG<br>n = 165 RH	75:25 real	97.1 (97.0 – 97.3)	96.1 (95.9 – 96.3)	93.8 (93.5 – 94.1)	96.4 (95.9 – 96.9)
	75:25 random	66.9 (65.5 – 68.2)	44.2 (42.8 – 45.5)	58.5 (57.5 – 59.4)	43.9 (42.2 – 45.6)
	χ^2	30.9, $p < 0.001$	64.3, $p < 0.001$	34.3, $p < 0.001$	65.8, $p < 0.001$
B. grahamii (V)<br>n = 193 all sites<br>n = 108 MW<br>n = 58 MFG<br>n = 27 RH	75:25 real	85.0 (84.6 – 85.4)	87.1 (86.6 – 87.6)	72.5 (71.5 – 73.5)	NA
	75:25 random	45.9 (45.1 – 46.1)	66.6 (65.8 – 67.3)	35.4 (32.9 – 37.9)	NA
	χ^2	33.8, $p < 0.001$	11.8, $p < 0.001$	27.7, $p < 0.001$	NA
B. taylorii (V)<br>n = 345 all sites<br>n = 94 MW<br>n = 110 MFG<br>n = 124 RH	75:25 real	95.5 (95.3 – 95.7)	86.9 (86.4 – 87.5)	94.8 (94.4 – 95.1)	NA
	75:25 random	33.6 (32.5 – 34.8)	39.5 (37.2 – 41.7)	84.2 (83.7 – 84.8)	NA
	χ^2	83.7, $p < 0.001$	48.3, $p < 0.001$	59.8, $p < 0.001$	NA
B. birtlesii (V)<br>n = 121 all sites<br>n = 72 MW<br>n = 60 MFG<br>n = 18 RH	75:25 real	NA	NA	NA	NA
	75:25 random	NA	NA	NA	NA
	χ^2	NA	NA	NA	NA
