# Supplementary material for: Multihost Bartonella parasites display covert host specificity even when transmitted by generalist vectors
Source: J Anim Ecol. 2016 Aug 16;85(6):1442–52. doi: 10.1111/1365-2656.12568 (PMC5082552; doi:10.1111/1365-2656.12568)
Supplement: Supplementary file 10 — Table S6. As Table S5, but using a reduced data set that includes only a single record of a particular Bartonella species or pITS variant for each individual. [file JANE-85-1442-s010.pdf]

Table S6 As Table S5, but using a reduced data set that includes only a single record of a particular *Bartonella* species or pITS variant for each individual, to account for the possibility of non-independence of data arising from sampling of some individuals on multiple occasions. The results agree with those using the full data set, therefore indicating that the pseudoreplication arising from multiple captures per individual does not influence the patterns of host-specificity identified.

**Data used**	**Test**	**% Prediction success**			
		**All Sites**	**MW**	**MFG**	**RH**
All *Bartonella* spp. (S)<br>n = 1106 all sites<br>n = 473 MW<br>n = 357 MFG<br>n = 276 RH	75:25 real	76.2 (75.8 – 76.5)	86.6 (85.9 – 87.2)	64.4 (63.7 – 65.1)	67.3 (66.7 – 67.8)
	75:25 random	30.7 (29.4 – 32.0)	34.4 (30.6 – 38.2)	44.2 (43.5 – 45.0)	50.4 (48.7 – 52.0)
	χ^2	41.6, $p < 0.001$	57.0, $p < 0.001$	8.2, $p = 0.004$	5.9, $p = 0.015$
Shared *Bartonella* spp. (S)<br>n = 933 all sites<br>n = 409 MW<br>n = 298 MFG<br>n = 226 RH	75:25 real	67.4 (67.0 – 67.8)	82.4 (81.8 – 83.1)	57.0 (56.0 – 57.9)	49.5 (48.8 – 50.2)
	75:25 random	18.1 (17.3 – 18.9)	25.3 (24.0 – 26.6)	53.6 (53.1 – 54.2)	32.2 (30.7 – 33.8)
	χ^2	51.4, $p < 0.001$	65.6, $p < 0.001$	0.23, $p = 0.63$	6.19, $p = 0.013$
All *Bartonella* spp. (V)<br>n = 710 all sites<br>n = 289 MW<br>n = 233 MFG<br>n = 180 RH	75:25 real	97.4 (97.3 – 97.5)	96.6 (96.4 – 96.8)	94.4 (94.0 – 94.7)	96.5 (95.9 – 97.0)
	75:25 random	70.9 (69.7 – 72.1)	47.4 (46.2 – 48.7)	60.7 (59.4 – 61.9)	60.9 (59.2 – 62.6)
	χ^2	26.3, $p < 0.001$	60.0, $p < 0.001$	32.6, $p < 0.001$	37.8, $p < 0.001$
Shared *Bartonella* spp. (V)<br>n = 573 all sites<br>n = 231 MW<br>n = 192 MFG<br>n = 142 RH	75:25 real	96.7 (96.4 – 97.0)	95.5 (95.2 – 95.8)	93.2 (92.3 – 94.0)	95.9 (95.4 – 96.5)
	75:25 random	69.9 (67.4 – 72.3)	42.9 (41.7 – 44.0)	64.2 (60.1 – 68.3)	52.7 (50.7 – 54.6)
	χ^2	25.8, $p < 0.001$	64.9, $p < 0.001$	25.1, $p < 0.001$	48.9, $p < 0.001$
B. grahamii (V)<br>n = 172 all sites<br>n = 98 MW<br>n = 50 MFG<br>n = 22 RH	75:25 real	82.3 (81.8 – 82.8)	85.3 (84.7 – 85.9)	67.8 (66.7 – 69.0)	NA
	75:25 random	52.0 (512 – 52.9)	71.3 (70.5 – 72.1)	44.6 (36.0 – 53.1)	NA
	χ^2	20.8, $p < 0.001$	5.77, $p < 0.001$	10.9, $p < 0.001$	NA
B. taylorii (V)<br>n = 293 all sites<br>n = 88 MW<br>n = 97 MFG<br>n = 103 RH	75:25 real	95.2 (94.7 – 95.7)	86.5 (86.0 – 87.1)	94.3 (93.8 – 94.8)	NA
	75:25 random	34.1 (33.2 – 34.9)	40.7 (37.9 – 43.5)	84.2 (81.2 – 87.2)	NA
	χ^2	81.7, $p < 0.001$	53.7, $p < 0.001$	5.3, $p < 0.001$	NA
B. birtlesii (V)<br>n = 108 all sites<br>n = 45 MW<br>n = 45 MFG<br>n = 17 RH	75:25 real	NA	NA	NA	NA
	75:25 random	NA	NA	NA	NA
	χ^2	NA	NA	NA	NA
