# Supplementary material for: Multihost Bartonella parasites display covert host specificity even when transmitted by generalist vectors
Source: J Anim Ecol. 2016 Aug 16;85(6):1442–52. doi: 10.1111/1365-2656.12568 (PMC5082552; doi:10.1111/1365-2656.12568)
Supplement: Supplementary file 11 — Table S7. Results of linear discriminant analyses that modelled host species identity based on the morphological identification of the flea species collected from them. [file JANE-85-1442-s011.pdf]

Table S7 Results of linear discriminant analyses that modelled host species identity based on the morphological identification of the flea species collected from them. Percentage successful prediction of host species is given for each model, where the model was first trained using 75% of real host-parasite associations (75:25 real) and then using 75% of randomised host-parasite associations (75:25 random). Mean percentage success of 1000 iterations is given, with 95% confidence intervals in brackets, and the success rates of models trained on the real data versus randomised data were compared using a Chi squared analysis. Results are first shown for all available data, then, as multiple flea specimens were sometimes collected from the same individual, the analysis was conducted using a reduced data set to account for potential pseudoreplication of host-vector associations. In the latter case, if multiple specimens of the same flea species were collected from an individual rodent, either at the same sampling occasion or on different sampling occasions, this host species-flea species association was included only once in the data set. Flea species appearing fewer than 5 times in the data set were omitted from the analysis and this is indicated in the table if relevant.

**Data used**	**Test**	**% Prediction success**
All host-flea data<br>*n* = 911<br>Not including *P. sorcis*	75:25 real	30.4 (30.1 – 30.8)
	75:25 random	30.7 (30.3 – 31.2)
	χ^2	0.212, $p = 0.88$
Host-flea data without pseudoreplication<br>*n* = 708<br>Not including *P. sorcis* or *T. p. poppei*	75:25 real	28.6 (28.2 – 29.0)
	75:25 random	30.1 (29.6 – 30.5)
	χ^2	5.4, $p = 0.46$
