# Supplementary material for: Multihost Bartonella parasites display covert host specificity even when transmitted by generalist vectors
Source: J Anim Ecol. 2016 Aug 16;85(6):1442–52. doi: 10.1111/1365-2656.12568 (PMC5082552; doi:10.1111/1365-2656.12568)
Supplement: Supplementary file 12 — Table S8. The species identity and Bartonella infection status of fleas collected from rodents. [file JANE-85-1442-s012.pdf]

Table S8 Number of fleas collected, number and % of *Bartonella*-positive fleas, and number and % of positive specimens for which *Bartonella* parasites were characterised, for each flea species. The number of each pITS *Bartonella* variant detected in each flea species is given. Numbers in brackets are the number of each pITS *Bartonella* variant detected in each flea species according to data from MFG and RH in 2012 only. Wood-mouse exclusive variants are highlighted in yellow; bank-vole exclusive variants are highlighted in green; variants previously detected in both rodents ("shared") are highlighted in purple; variants detected for the first time in this study, and therefore so far only detected in fleas, are highlighted in grey.

		A p. mustelae		***C.n. vulgaris***		***H.t. talpae***		***M. turbidus***		***P. sorcis***		***R. pentacantha***		***T.p. poppei***	
# specimens screened		127		541		26		137		2		44		4	
# *Bartonella*-positive		68 (54%)		273 (50%)		13 (50%)		97 (71%)		1 (50%)		8 (18%)		0 (0%)	
# *Bartonella* parasites characterised genetically		57 (84%)		229 (84%)		7 (54%)		82 (85%)		1 (100%)		6 (75%)		NA	
***Bartonella* species**	**pITS variant**	**WM**	**BV**	**WM**	**BV**	**WM**	**BV**	**WM**	**BV**	**WM**	**BV**	**WM**	**BV**	**WM**	**BV**
B. doshiae	doshiae-1			1 (1)			1		1						
	doshiae-2												1		
B. doshiae-like	doshiae-like-1			7 (3)	2 (1)										
B. rochalimae-like	rochalimae-like-1	1	15 (2)		1			3	31						
BGA	BGA-1	1		13 (3)	3			1							
B. birtlesii	birtlesii-5			9 (3)		2 (1)						1 (1)			
	birtlesii-7			2											
	birtlesii-1			1	11 (1)				1				1		
	birtlesii-8			4 (1)	9 (1)										
	birtlesii-9			2	4										
B. grahamii	grahamii-1	1	13 (1)	2 (1)	6 (1)			2 (1)	16						
	grahamii-2	1	7		10	1		2	14						
	grahamii-4			2				1							
	grahamii-5	2 (2)	2		1										
	grahamii-6	1						1 (1)	1						
	grahamii-7							1 (1)							
	grahamii-8			1											
B. taylorii	taylorii-2	1		1	7 (1)								1		
	taylorii-6			5	2										
	taylorii-7			1	1										
	taylorii-8	1		11	3 (1)	1									
	taylorii-9			1 (1)	1										
	taylorii-10			2											
	taylorii-3	1	7 (1)	20 (1)	48 (1)		2 (1)		4						
	taylorii-4	2		14 (2)	4 (1)					1					
	taylorii-5			1	9				1			1	1		
	taylorii-11			1 (1)											
	taylorii-12			2	3										
B. tribocorum	tribocorum-1							2							
Unknown	unknown-1		1												
