# Supplementary material for: Multihost Bartonella parasites display covert host specificity even when transmitted by generalist vectors
Source: J Anim Ecol. 2016 Aug 16;85(6):1442–52. doi: 10.1111/1365-2656.12568 (PMC5082552; doi:10.1111/1365-2656.12568)
Supplement: Supplementary file 13 — Table S9. GenBank accession numbers of the ten Bartonella partial 16S‐23S ITS sequence variants detected in fleas only. [file JANE-85-1442-s013.pdf]

Table S9 The GenBank accession numbers of the ten *Bartonella* partial 16S-23S ITS sequence types detected in fleas only in this study. Sequence types are grouped into *Bartonella* species groups based on their closest match to existing named *Bartonella* species within GenBank.

***Bartonella* species**	**pITS variant**	**BLAST accession number**
B. grahamii	grahamii-6	KU589247
	grahamii-7	KU589248
	grahamii-8	KU589249
B. taylorii	taylorii-11	KU589250
	taylorii-12	KU589251
B. birtlesii	birtlesii-8	KU589252
	birtlesii-9	KU589253
B. doshiae	doshiae-2	KU589254
Unknown	unknown-1	KU589255
B. tribocorum	tribocorum-1	KU589256
