# Supplementary material for: Multihost Bartonella parasites display covert host specificity even when transmitted by generalist vectors
Source: J Anim Ecol. 2016 Aug 16;85(6):1442–52. doi: 10.1111/1365-2656.12568 (PMC5082552; doi:10.1111/1365-2656.12568)
Supplement: Supplementary file 14 — Table S10. Results of linear discriminant analyses that modelled host species identity based on the variant of Bartonella carried by fleas collected from them. [file JANE-85-1442-s014.pdf]

Table S10 Results of linear discriminant analyses that modelled host species identity based on the variant of *Bartonella* carried by fleas collected from them. Percentage successful prediction of host species is given for each model, where the model was first trained using 75% of real host-parasite associations (75:25 real) and then using 75% of randomised host-parasite associations (75:25 random). Mean percentage success of 1000 iterations is given, with 95% confidence intervals in brackets, and the success rates of models trained on the real data versus randomised data were compared using a Chi squared analysis. An LDA was performed using data on the *Bartonella* infections of all flea species combined and then using data on the *Bartonella* infections of *C. n. vulgaris* only. Analyses were not possible using data from other flea species independently as most variants were found <5 times in these other species and models could not be constructed with such a small amount of data (Table S8). Models were also constructed using a reduced data set that included only a single record of a *Bartonella* variant in fleas from a given host individual, even if multiple fleas harbouring the same *Bartonella* variant were collected from the same individual. *Bartonella* variants occurring in fleas fewer than 5 times in the data set were omitted from the analysis and this is indicated in the table if relevant.

Data used	Test	% Prediction success
All flea-*Bartonella* data, all flea species $n = 333$	75:25 real	85.3 (84.8 – 85.8)
	75:25 random	49.8 (48.8 – 50.9)
	χ^2	28.7, $p < 0.001$
Flea-*Bartonella* data without pseudoreplication, all flea species $n = 287$	75:25 real	83.6 (83.0 – 84.1)
	75:25 random	50.5 (49.4 – 51.6)
	χ^2	24.8, $p < 0.001$
All flea-*Bartonella* data, *C. n. vularis* only $n = 189$ Not including fleas infected with variants Type 08 or 10	75:25 real	86.4 (85.8 – 87.0)
	75:25 random	55.9 (54.4 – 57.3)
	χ^2	21.4, $p < 0.001$
Flea-*Bartonella* data without pseudoreplication, *C. n. vulgaris* only $n = 174$ Not including fleas infected with variants Type 08 or 10	75:25 real	84.8 (84.2 – 85.5)
	75:25 random	57.5 (55.8 – 59.1)
	χ^2	18.2, $p < 0.001$
