# Supplementary material for: Multihost Bartonella parasites display covert host specificity even when transmitted by generalist vectors
Source: J Anim Ecol. 2016 Aug 16;85(6):1442–52. doi: 10.1111/1365-2656.12568 (PMC5082552; doi:10.1111/1365-2656.12568)
Supplement: Supplementary file 15 — Table S11. Bartonella species submissions in GenBank with which each pITS variant in this study shares highest and second highest similarity. [file JANE-85-1442-s015.pdf]

Table S11 The accession numbers and percentage similarity of *Bartonella* species submissions in GenBank with which each pITS variant in this study shares the highest and second highest similarity.

pITS variant	pITS length (bp)	GenBank submission with highest similarity		GenBank submission with second highest similarity	
		Species	Accession # and % similarity	Species	Accession # and % similarity
doshiae-1	292	*B. doshiae*	AJ269786.1 (99%)	*B. doshiae*-like	AJ269792.1* (89%)
doshiae-2	308	*B. doshiae*	AF442954.1 (99%)	*B. doshiae*-like	AJ269792.1* (85%)
doshiae-like-1	293	*B. doshiae*-like*	AJ269792.1 (100%)	*B. doshiae*	AJ269786.1 (89%)
rochalimae-like-1	461	Candidatus *B. rudakovii*	EF682087.1 (100%)	*B. rochalimae*	KU292577.1 (85%)
BGA-1	466	BGA+	DQ155376.1 (100%)	Candidatus *B. rudakovii*	EF682087.1 (89%)
birtlesii-1	370	*B. birtlesii* (sp. N40)†	AJ269791.1 (97%)	*B. taylorii*	JN810861.1 (96%)
birtlesii-2	370	*B. birtlesii* (sp. N40)†	AJ269787.1 (99%)	*B. taylorii*	JN810861.1 (95%)
birtlesii-3	370	*B. birtlesii* (sp. N40)†	AJ269791.1 (100%)	*B. taylorii*	JN810861.1 (98%)
birtlesii-4	351	*B. birtlesii*	KC907380.1 (100%)	*B. taylorii*	AJ269788.1 (98%)
birtlesii-5	370	*B. birtlesii* (sp. N40)†	AJ269787.1 (100%)	*B. taylorii*	JN810861.1 (96%)
birtlesii-6	391	*B. birtlesii* (sp. N40)†	AJ269787.1 (95%)	*B. taylorii*	JN810861.1 (92%)
birtlesii-7	389	*B. birtlesii* (sp. N40)†	AJ269787.1 (94%)	*B. taylorii*	JN810861.1 (91%)
birtlesii-8	371	*B. birtlesii* (sp. N40)†	AJ269791.1 (98%)	*B. taylorii*	JN810861.1 (96%)
birtlesii-9	370	*B. birtlesii* (sp. N40)†	AJ269791.1 (98%)	*B. taylorii*	JN810861.1 (97%)
grahamii-1	314	*B. grahamii*	JN810855.1 (99%)	*B. queenslandensis*	EU111768.1 (82%)
grahamii-2	315	*B. grahamii*	JN810855.1 (99%)	*B. queenslandensis*	EU111768.1 (82%)
grahamii-3	315	*B. grahamii*	JN810855.1 (99%)	*B. queenslandensis*	EU111768.1 (82%)
grahamii-4	315	*B. grahamii*	AJ269790.1 (100%)	*B. queenslandensis*	EU111768.1 (82%)
grahamii-5	315	*B. grahamii*	JN810855.1 (100%)	*B. queenslandensis*	EU111768.1 (82%)
grahamii-6	315	*B. grahamii*	AJ269790.1 (99%)	*B. queenslandensis*	EU111768.1 (83%)
grahamii-7	315	*B. grahamii*	AJ269790.1 (99%)	*B. queenslandensis*	EU111768.1 (82%)
grahamii-8	315	*B. grahamii*	JN810855.1 (99%)	*B. queenslandensis*	EU111768.1 (82%)
taylorii-1	352	*B. taylorii*	AJ269788.1 (99%)	*B. birtlesii* (sp. N40)†	AJ269791.1 (94%)
taylorii-2	352	*B. taylorii*	AJ269788.1 (99%)	*B. birtlesii* (sp. N40)†	AJ269791.1 (94%)
taylorii-3	352	*B. taylorii*	AJ269788.1 (99%)	*B. birtlesii* (sp. N40)†	AJ269791.1 (94%)
taylorii-4	352	*B. taylorii*	AJ269788.1 (100%)	*B. birtlesii* (sp. N40)†	AJ269791.1 (94%)
taylorii-5	352	*B. taylorii*	AJ269788.1 (98%)	*B. birtlesii* (sp. N40)†	AJ269791.1 (94%)
taylorii-6	352	*B. taylorii*	AJ269788.1 (99%)	*B. birtlesii* (sp. N40)†	AJ269791.1 (94%)
taylorii-7	353	*B. taylorii*	AJ269788.1 (99%)	*B. birtlesii* (sp. N40)†	AJ269791.1 (94%)
taylorii-8	351	*B. taylorii*	AJ269784.1 (100%)	*B. birtlesii* (sp. N40)†	AJ269791.1 (93%)
taylorii-9	351	*B. taylorii*	AJ269784.1 (99%)	*B. birtlesii* (sp. N40)†	AJ269791.1 (93%)
taylorii-10	352	*B. taylorii*	AJ269788.1 (99%)	*B. birtlesii* (sp. N40)†	AJ269791.1 (94%)
taylorii-11	349	*B. taylorii*	AJ269784.1 (99%)	*B. birtlesii* (sp. N40)†	AJ269791.1 (92%)
taylorii-12	352	*B. taylorii*	AJ269788.1 (98%)	*B. birtlesii* (sp. N40)†	AJ269791.1 (93%)
tribocorum-1	387	*B. tribocorum*	HG969192.1 (100%)	*B. elizabethae*	L35103.1 (81%)
unknown-1	459	BGA+	DQ155376.1 (92%)	Candidatus *B. rudakovii*	EF682087.1 (90%)

* Sample "wbs11" was subsequently referred to in their papers and others (e.g. Telfer *et al.*, 2007a) as *B. doshiae*-like.
+ Pers. Comms. with S. Telfer that this sequence matches that previously referred to as BGA (Telfer *et al.*, 2007a).
† Pers. Comms. with R. Birtles confirmed that "N40" was subsequently named *B. birtlesii*.
