# Supplementary material for: Multihost Bartonella parasites display covert host specificity even when transmitted by generalist vectors
Source: J Anim Ecol. 2016 Aug 16;85(6):1442–52. doi: 10.1111/1365-2656.12568 (PMC5082552; doi:10.1111/1365-2656.12568)
Supplement: Supplementary file 16 — Table S12. Proportions of trap locations at which both wood mice and bank voles were captured during each trapping session at each site. [file JANE-85-1442-s016.pdf]

Table S12 The number of trap locations during each trapping session per site where at least one rodent was captured (“occupied” locations; occ) and the number (in **bold**) and proportion (in *italics*) of these trap locations where both wood mice and bank voles were captured (“multispecies” locations; ms). The median proportion of multi-species locations across all sites and sessions was 0.2. The total numbers of traps deployed on each trapping grid are shown in brackets.

Site	Grid		Session 1	Session 2	Session 3	Session 4	Session 5	Session 6	Session 7	Session 8
Manor Wood	MW1<br>(128 traps)	occ:<br>ms:	27<br>**5**<br>*0.19*	36<br>**7**<br>*0.19*	32<br>**7**<br>*0.22*	49<br>**19**<br>*0.39*	29<br>**7**<br>*0.24*	21<br>**2**<br>*0.10*	27<br>**2**<br>*0.07*	26<br>**2**<br>*0.08*
	MW2<br>(128 traps)	occ:<br>ms:	NA	46<br>**9**<br>*0.20*	46<br>**14**<br>*0.30*	55<br>**12**<br>*0.22*	29<br>**8**<br>*0.28*	18<br>**0**<br>*0*	28<br>**3**<br>*0.11*	12<br>**0**<br>*0*
	MW3<br>(72 traps)	occ:<br>ms:	24<br>**3**<br>0.13	29<br>**8**<br>*0.28*	35<br>**20**<br>*0.57*	35<br>**23**<br>*0.66*	14<br>**2**<br>*0.14*	21<br>**4**<br>*0.19*	17<br>**6**<br>*0.36*	NA
	MW4<br>(72 traps)	occ:<br>ms:	16<br>**3**<br>*0.19*	27<br>**4**<br>*0.15*	29<br>**5**<br>*0.17*	35<br>**19**<br>*0.54*	27<br>**10**<br>*0.37*	15<br>**2**<br>*0.13*	22<br>**2**<br>*0.09*	NA
Maresfield & Gordale	Gordale<br>(72 traps)	occ:<br>ms:	28<br>**11**<br>*0.39*	34<br>**9**<br>*0.26*	13<br>**2**<br>*0.15*	21<br>**6**<br>*0.29*	31<br>**4**<br>*0.13*	34<br>**10**<br>*0.29*	26<br>**4**<br>*0.15*	20<br>**3**<br>*0.15*
	MF1<br>(72 traps)	occ:<br>ms:	33<br>**18**<br>*0.55*	29<br>**11**<br>*0.38*	19<br>**3**<br>*0.16*	23<br>**7**<br>*0.30*	26<br>**4**<br>*0.15*	30<br>**8**<br>*0.27*	29<br>**3**<br>*0.10*	19<br>**1**<br>*0.05*
	MF2<br>(72 traps)	occ:<br>ms:	34<br>20<br>*0.59*	33<br>11<br>*0.33*	28<br>9<br>*0.32*	25<br>11<br>*0.44*	33<br>6<br>*0.18*	31<br>8<br>*0.26*	31<br>9<br>*0.29*	24<br>2<br>*0.08*
Rode Hall	RH2<br>(72 traps)	occ:<br>ms:	15<br>**3**<br>*0.2*	21<br>**7**<br>*0.33*	26<br>**7**<br>*0.27*	25<br>**6**<br>*0.24*	28<br>**5**<br>*0.18*	30<br>**4**<br>*0.13*	17<br>**2**<br>*0.12*	10<br>**2**<br>*0.2*
	RH3<br>(72 traps)	occ:<br>ms:	29<br>**9**<br>*0.31*	35<br>**7**<br>*0.2*	31<br>**4**<br>*0.13*	25<br>**5**<br>*0.2*	28<br>**13**<br>*0.46*	34<br>**11**<br>*0.32*	23<br>**2**<br>*0.09*	21<br>**1**<br>*0.05*
	RH4<br>(72 traps)	occ:<br>ms:	25<br>**5**<br>*0.2*	20<br>**3**<br>*0.15*	16<br>**0**<br>*0*	27<br>**3**<br>*0.11*	26<br>**4**<br>*0.15*	29<br>**4**<br>*0.14*	23<br>**4**<br>*0.17*	23<br>**6**<br>*0.26*
