# Supplementary material for: Multihost Bartonella parasites display covert host specificity even when transmitted by generalist vectors
Source: J Anim Ecol. 2016 Aug 16;85(6):1442–52. doi: 10.1111/1365-2656.12568 (PMC5082552; doi:10.1111/1365-2656.12568)
Supplement: Supplementary file 17 — Table S13. Comparison of the Bartonella pITS variants found in individual fleas and the variants found in the rodent hosts from which each flea was collected. [file JANE-85-1442-s017.pdf]

Table S13 The *Bartonella* pITS variants found in individual fleas and the *Bartonella* pITS variants found in the rodent hosts from which each flea was collected. Data are limited as the only year in which both rodents and fleas were screened for *Bartonella* DNA was 2012, and in addition, only a subset of the pITS amplicons from rodents was sequenced. Thus there were only 8 *Bartonella*-positive fleas collected from bank voles and 15 from wood mice for which data on the *Bartonella* variants carried by the rodent from which they were collected are also available.

Site	Flea species	*Bartonella* variant in flea	Rodent species	*Bartonella* variant in rodent blood	Variant match?
RH	C. n. vulgaris	birtlesii-8	Bank Vole	rochalimae-like-1	no
RH	A. p. mustelae	rochalimae-like-1	Bank Vole	taylorii-3	no
MFG	C. n. vulgaris	taylorii-8	Bank Vole	rudakovii-1	no
RH	C. n. vulgaris	taylorii-2	Bank Vole	taylorii-2	yes
RH	C. n. vulgaris	taylorii-3	Bank Vole	taylorii-3	yes
RH	H. t. talpae	taylorii-3	Bank Vole	taylorii-3	yes
RH	A. p. mustelae	taylorii-3	Bank Vole	taylorii-3	yes
MFG	C. n. vulgaris	grahamii-1	Bank Vole	grahamii-1	yes
RH	M. turbidus	grahamii-6	Wood Mouse	BGA-1	no
MFG	M. turbidus	grahamii-1	Wood Mouse	doshiae-like-1	no
MFG	A. p. mustelae	grahamii-5	Wood Mouse	birtlesii-5	no
MFG	C. n. vulgaris	grahamii-1	Wood Mouse	birtlesii-2	no
MFG	C. n. vulgaris	doshiae-1	Wood Mouse	taylorii-4	no
MFG	C. n. vulgaris	taylorii-9	Wood Mouse	grahamii-5	no
MFG	C. n. vulgaris	taylorii-11	Wood Mouse	taylorii-5	no
MFG	C. n. vulgaris	doshiae-like-1	Wood Mouse	taylorii-8	no
MFG	C. n. vulgaris	BGA-1	Wood Mouse	taylorii-9	no
RH	C. n. vulgaris	BGA-1	Wood Mouse	BGA-1	yes
MFG	C. n. vulgaris	doshiae-like-1	Wood Mouse	doshiae-like-1	yes
MFG	C. n. vulgaris	doshiae-like-1	Wood Mouse	doshiae-like-1	yes
MFG	A. p. mustelae	grahamii-5	Wood Mouse	grahamii-5	yes
MFG	R. pentacantha	birtlesii-5	Wood Mouse	birtlesii-5	yes
MFG	C. n. vulgaris	taylorii-4	Wood Mouse	taylorii-4	yes
